# Supplementary material for: Construction of a trio-based structural variation panel utilizing activated T lymphocytes and long-read sequencing technology
Source: Commun Biol. 2022 Sep 20;5:991. doi: 10.1038/s42003-022-03953-1 (PMC9489684; doi:10.1038/s42003-022-03953-1)
Supplement: Supplementary file 1 — Supplementary Information [file 42003_2022_3953_MOESM1_ESM.pdf]

Supplementary Information

Construction of a trio-based structural variation panel utilizing activated T lymphocytes and long-read sequencing technology

Authors

Akihito Otsuki[#], Yasunobu Okamura[#], Noriko Ishida, Shu Tadaka, Jun Takayama, Kazuki Kumada, Junko Kawashima, Keiko Taguchi, Naoko Minegishi, Shinichi Kuriyama, Gen Tamiya, Kengo Kinoshita, Fumiki Katsuoka, and Masayuki Yamamoto*

# These authors contributed equally

* Corresponding author

Supplementary Figure 1. Optimization of read length and sequencing yield for population scale analysis.

Supplementary Figure 2. Benchmark analysis using three pairs of donor-matched data.

Supplementary Figure 3. Sequencing statistics from individual runs.

Supplementary Figure 4. Relationship between N50 and SV detection.

Supplementary Figure 5. Comparison of allele frequencies to published datasets.

Supplementary Figure 6. MIEs observed for each SV.

Supplementary Figure 7. Numbers of reads supporting SV calls.

Supplementary Figure 8. Distribution of insertion lengths in the *ATXN3* gene of different lengths in each individual.

Supplementary Figure 9. Trio-based WGS utilizing activated T cells enables us to effectively catalog SVs at the population scale.

Supplementary Figures

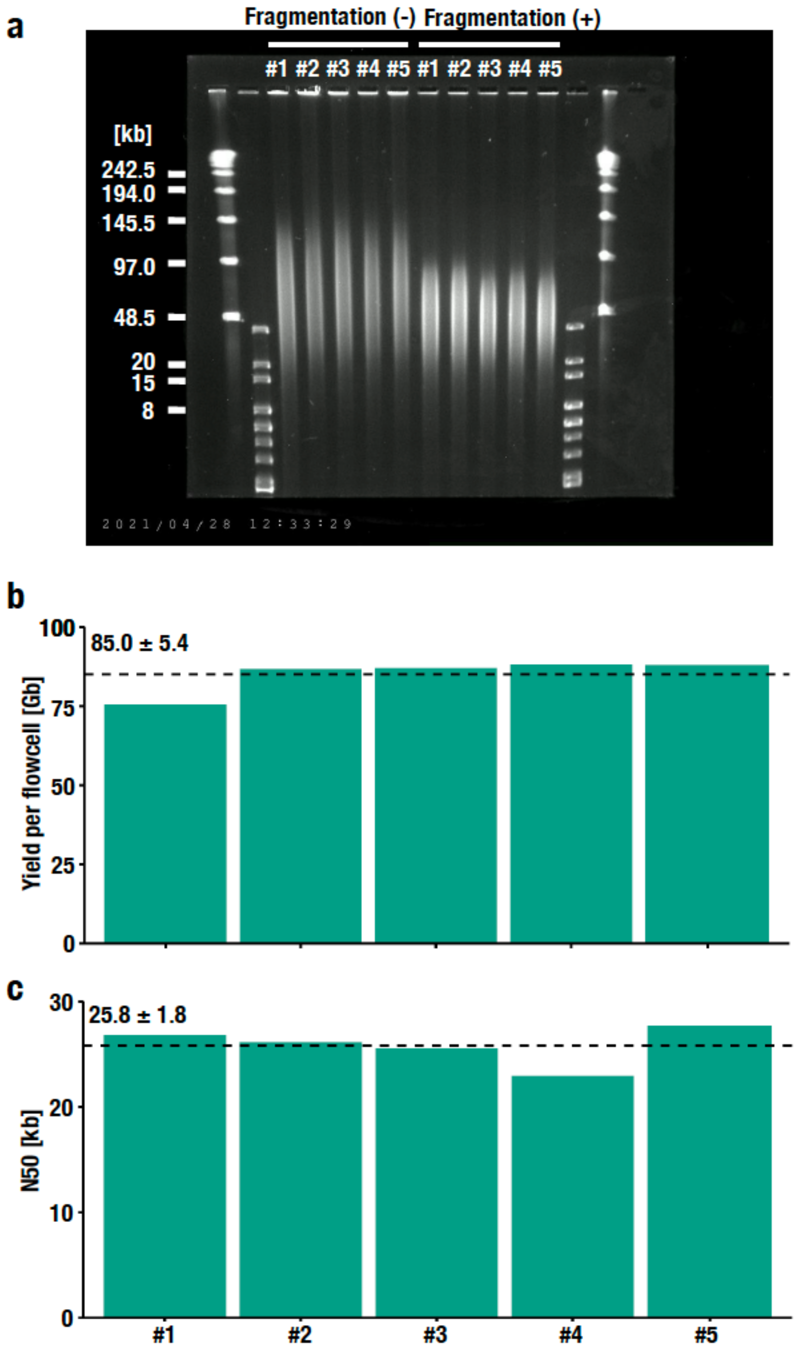

Supplementary Figure 1. Optimization of read length and sequencing yield for population scale analysis. a An uncropped image of Figure 1d. The image was stretched/compressed to fit the figure layout in Figure 1d. **b** and **c** Sequencing yield per flowcell (**b**) and N50 (**c**) using five independent samples (from #1 to #5 shown in Fig.

1d). The dotted lines show the mean values, and the mean ± S.D. are indicated in the panel.

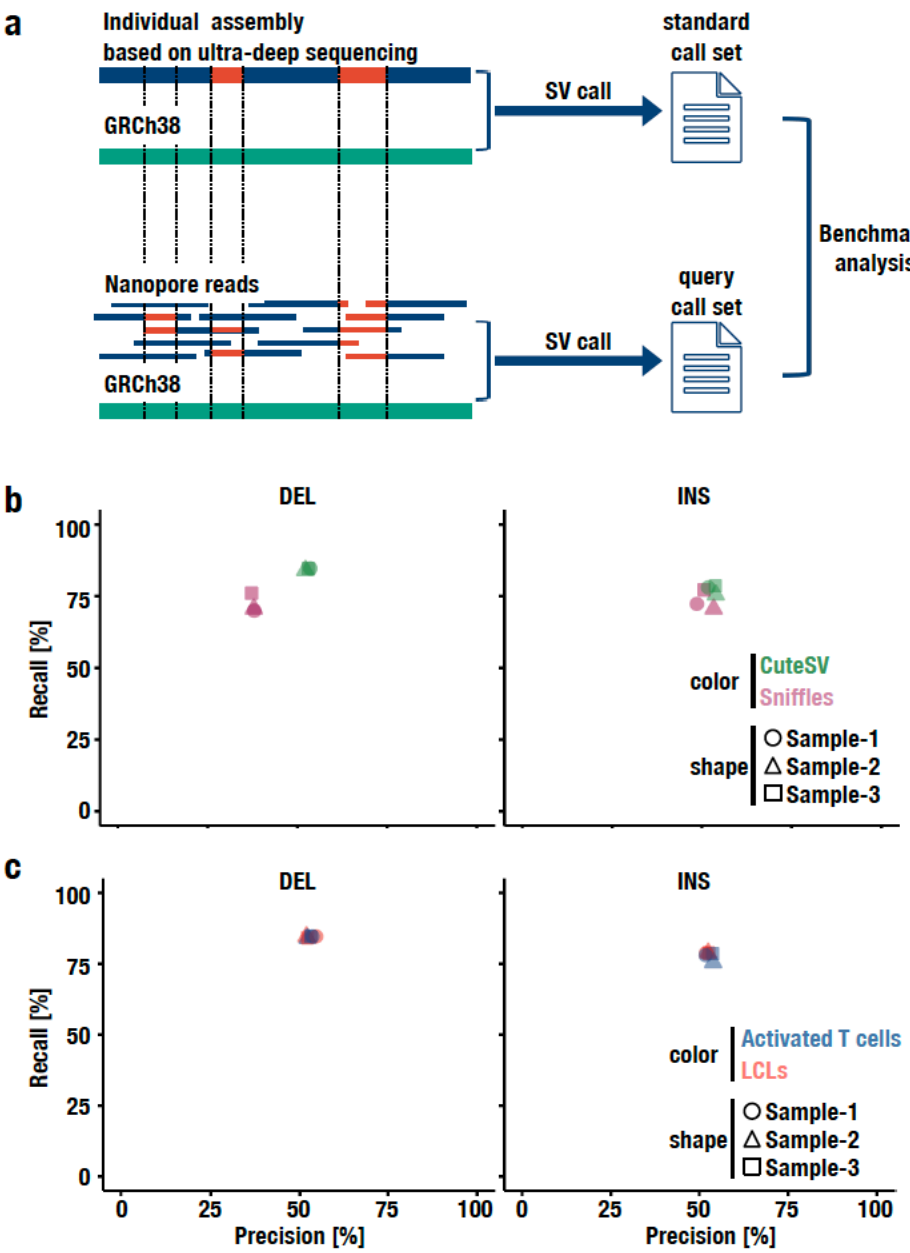

Supplementary Figure 2. Benchmark analysis using three pairs of donor-matched data. **a** Design of the benchmark analysis. We obtained three pairs of standard SV-call sets from high-contiguity assemblies with ultradeep sequencing and optical mapping data[41] and query call sets from nanopore sequencing data by comparing them to the GRCh38 reference genome. SVs are indicated in red. Precision and recall were calculated for each set of SV calls. Notably, the assemblies are pseudohaploid in which only one (randomly selected) allele of each heterozygous SV is retained. **b** Precision-recall plot of the SV calling software. The software is represented by color, whereas the samples are

represented by shapes as specified in the legend. **c** Precision-recall plot of DNA resources. The DNA resources are represented by color, whereas the samples are represented by shapes as specified in the legend.

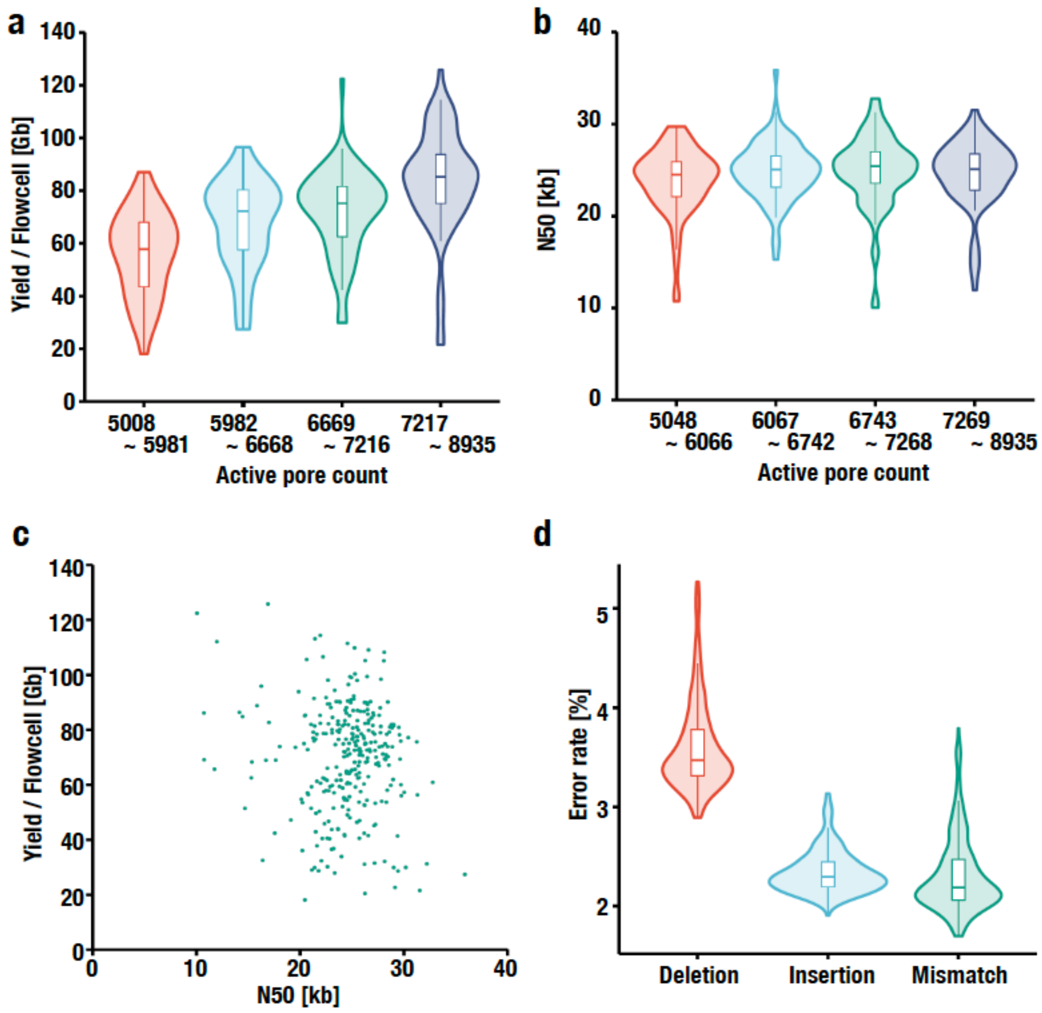

Supplementary Figure 3. Sequencing statistics from individual runs. a and b Relationship between the qualities of flowcells and the sequencing yield per flowcell **(a)** and between the qualities of flowcells and the N50 **(b)**. The qualities of flowcells are categorized according to the quartile of the active pore count. Each boxplot has a box that represents the interquartile range (IQR) and whiskers that extend 1.5 x IQR from the box edges. The median is shown in a horizontal line in the box. **c** Scatter plot showing the relationship between N50 and the sequencing yields per flowcell. **d** Error profile of nanopore sequencing. Each boxplot has a box that represents the interquartile range (IQR) and whiskers that extend 1.5 x IQR from the box edges. The median is shown in a horizontal line in the box.

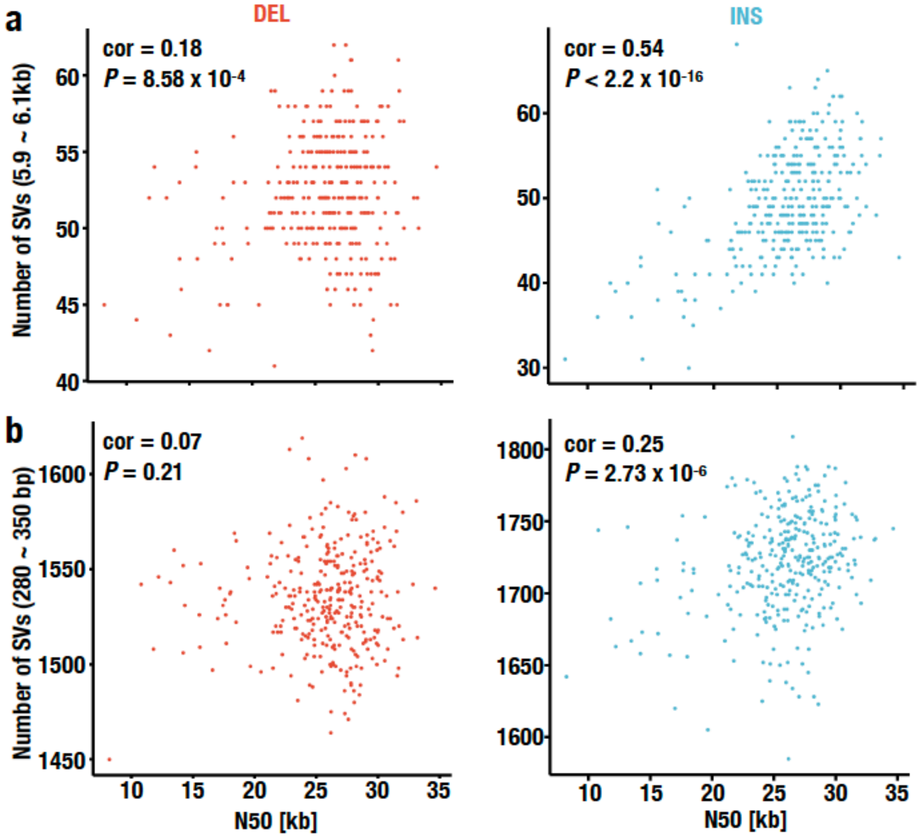

Supplementary Figure 4. Relationship between N50 and SV detection. a-b Scatter plot showing the relationship between the N50 length and the number of SVs within the range from 5.9 kb to 6.1 kb **(a)** and the number of SVs within the range from 280 bp to 350 bp **(b)**. The Pearson correlation coefficient (cor) and *P* values are shown.

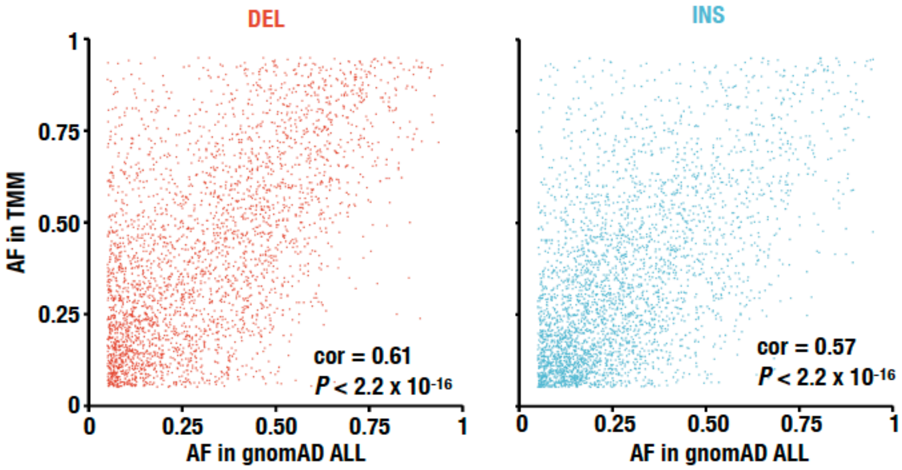

Supplementary Figure 5. Comparison of allele frequencies to published datasets. Scatterplot showing the allele frequencies estimated in all fractions of the gnomAD study (gnomAD ALL; x-axis) and this study (TMM; y-axis). The Pearson correlation coefficient (cor) and *P* values are shown.

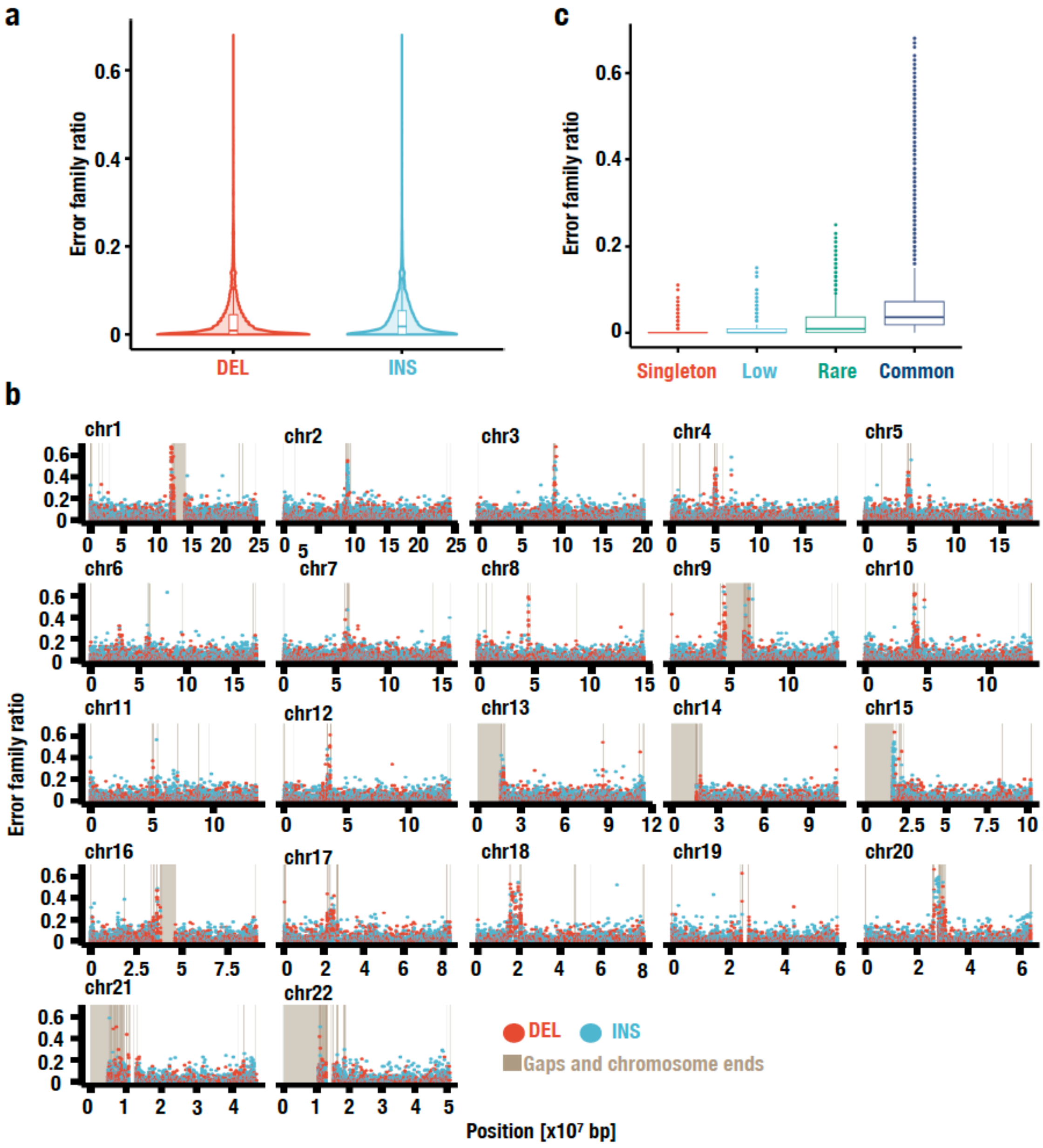

Supplementary Figure 6. MIEs observed for each SV. a Distribution of error family ratios of DELs (red) and INSs (blue). Each boxplot has a box that represents the interquartile range (IQR) and whiskers that extend 1.5 x IQR from the box edges. The median is shown in a horizontal line in the box. **b** Distribution of DELs (red) and INSs (blue) and the error family ratio at the chromosome scale. Positions of gaps and chromosome ends in GRCh38 are highlighted in brown. **c.** Distribution of error family ratios in each MAF category. Each boxplot includes a box that represents the interquartile

range (IQR) and whiskers that extend 1.5 x IQR from the box edges. The median is shown as a horizontal line in the box.

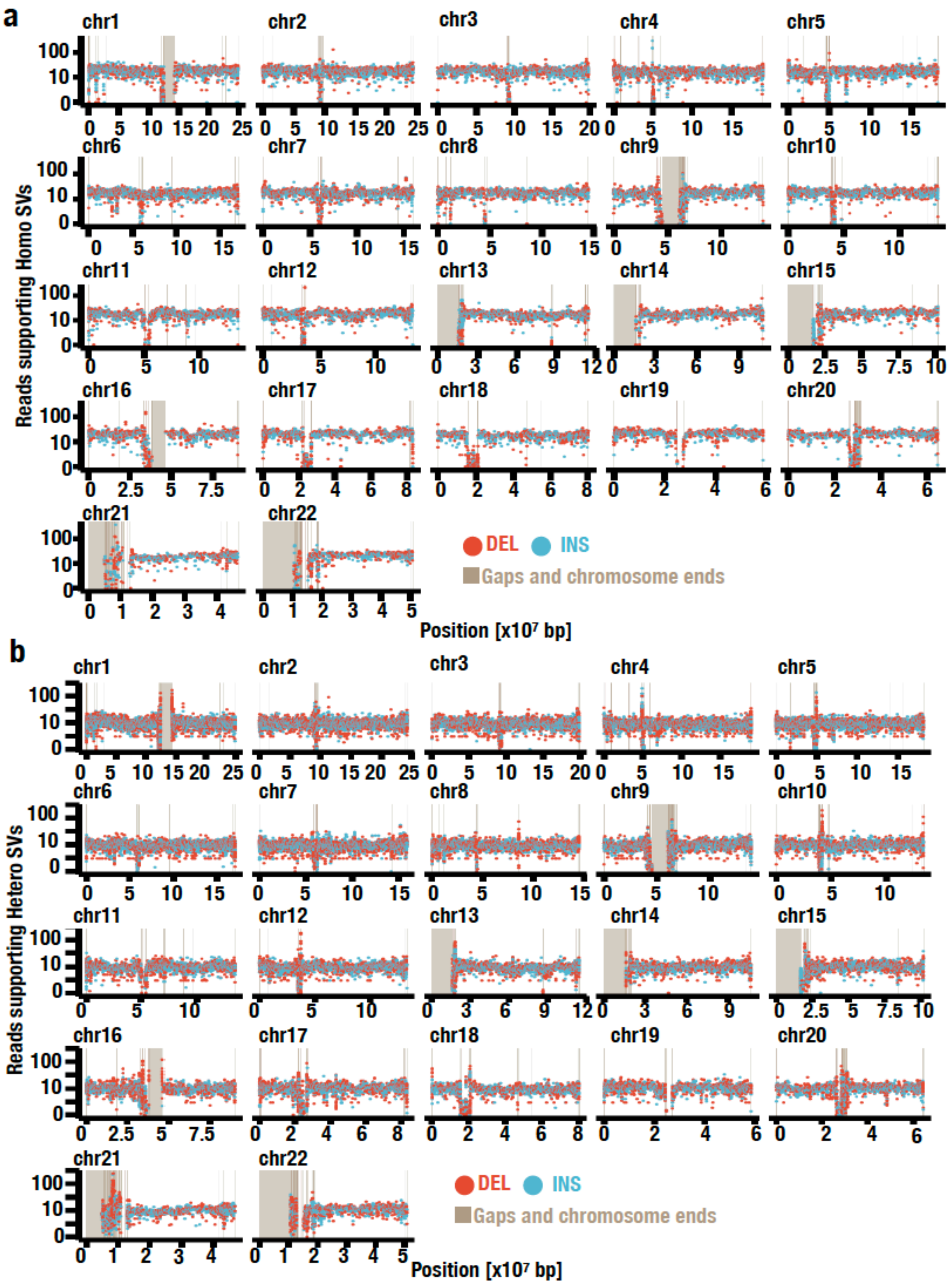

Supplementary Figure 7. Numbers of reads supporting SV calls. a and **b** Distribution of DELs (red) and INSs (blue) and the number of reads supporting variant calls for heterozygous (**a**) and homozygous. (**b**) Positions of gaps and chromosome ends in GRCh38 are highlighted in brown.

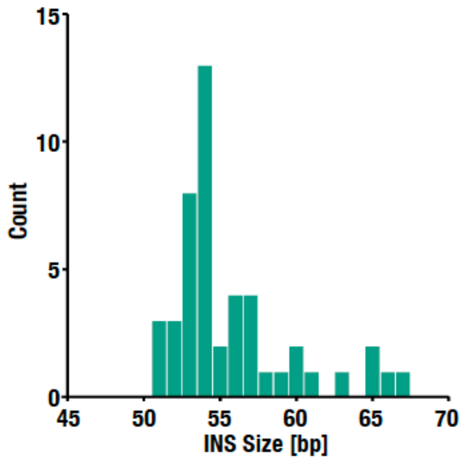

Supplementary Figure 8. Distribution of insertion lengths in the *ATXN3* gene of different lengths in each individual. Histogram showing the distribution of the size of the insertions in the *ATXN3* gene of different sizes.

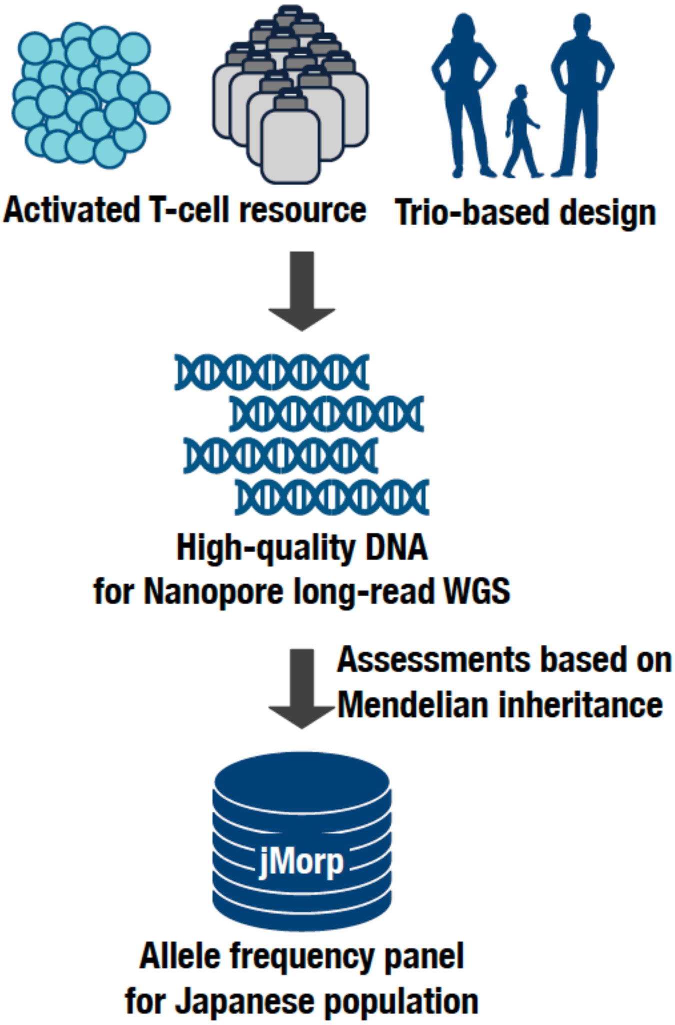

Supplementary Figure 9. Trio-based WGS utilizing activated T cells enables us to effectively catalog SVs at the population scale. We established the TMM Biobank, which stores activated T cells as a genomic DNA resource and is utilized to perform long read-based WGS to identify SVs and prepare an SV reference panel for the Japanese population. We also designed a trio-based analysis for the careful interpretation of the detected SVs using Mendelian error profiles. The SVs identified are publicly available on our website, jMorp (https://jmorp.megabank.tohoku.ac.jp/202112/).
