# Supplementary material for: Construction of a trio-based structural variation panel utilizing activated T lymphocytes and long-read sequencing technology
Source: Commun Biol. 2022 Sep 20;5:991. doi: 10.1038/s42003-022-03953-1 (PMC9489684; doi:10.1038/s42003-022-03953-1)
Supplement: Supplementary file 3 — Editorial Assessment Report [file 42003_2022_3953_MOESM3_ESM.pdf]

nature portfolio

GUIDED OPEN ACCESS

nature genetics | nature communications | communications biology

Contents of this report

1. **Manuscript details**: overview of your manuscript and the editorial team.
2. **Review synthesis**: summary of the reviewer reports provided by the editors.
3. **Editorial recommendation**: personalized evaluation and recommendation from all 3 journals.
4. **Annotated reviewer comments**: the referee reports with comments from the editors.
5. **Open research evaluation**: advice for adhering to best reproducibility practices.

About the editorial process

Because you selected the **Nature Portfolio Guided Open Access** option, your manuscript was assessed for suitability in three of our titles publishing high-quality work across the spectrum of genetics research: ***Nature Genetics***, ***Nature Communications***, and ***Communications Biology***. More information about Guided Open Access can be found here.

Collaborative editorial assessment

Your editorial team discussed the manuscript to determine its suitability for the Nature Portfolio Guided OA pilot. Our assessment of your manuscript takes into account several factors, including whether the work meets the **technical standard** of the Nature Portfolio and whether the findings are of **immediate significance** to the readership of at least one of the participating journals in the Nature Portfolio Guided Open Access genetics cluster.

Peer review

Experts were asked to evaluate the following aspects of your manuscript:

- **Novelty** in comparison to prior publications;
- **Likely audience** of researchers in terms of broad fields of study and size;
- **Potential impact** of the study on the immediate or wider research field;
- **Evidence** for the claims and whether additional experiments or analyses could feasibly strengthen the evidence;
- **Methodological detail** and whether the manuscript is reproducible as written;
- Appropriateness of the **literature review**.

Editorial evaluation of reviews

Your editorial team discussed the potential suitability of your manuscript for each of the participating journals. They then discussed the revisions necessary in order for the work to be published, keeping each journal's specific editorial criteria in mind.

Journals in the Nature portfolio will support authors wishing to transfer their reviews and (where reviewers agree) the reviewers' identities to journals outside of Springer Nature.
If you have any questions about review portability, please contact our editorial office at guidedoa@nature.com.

nature portfolio

GUIDED OPEN ACCESS

nature genetics | nature communications | communications biology

Manuscript details

Tracking number	Submission date	Decision date	Peer review type
GUIDEDOA-21-00277	Oct 3, 2021	Dec 7, 2021	Single-blind

Manuscript title

Construction of a trio-based structural variation panel utilizing activated T lymphocytes and long-read sequencing technology

Author details

Masayuki Yamamoto

Affiliation: Tohoku University

Editorial assessment team

**Primary editor**	**Michael Fletcher**<br>Home journal: ***Nature Genetics***<br>ORCID: **0000-0003-1589-7087**<br>Email: **michael.fletcher@nature.com**
**Other editors consulted**	**Margot Brandt**<br>Home journal: ***Nature Communications***<br>ORCID: **0000-0002-9434-794X**<br><br>**George Inglis**<br>Home journal: ***Communications Biology***<br>ORCID: **0000-0002-9069-5242**
**About your primary editor**	Michael Fletcher studied for his Ph.D. at Cancer Research UK's Cambridge Institute at the University of Cambridge in the laboratory of Bruce Ponder. His project used systems biology approaches to identify the functional mechanism by which FGFR2, a breast cancer risk locus identified using GWAS, exerts its effect. He then moved to Germany and was a postdoctoral fellow in the Molecular Genetics department of Peter

	Lichter at the German Cancer Research Center in Heidelberg, where he performed bioinformatics analysis to help characterize the epigenomic and master regulator landscapes of adult glioblastoma. He joined the journal in 2020.

Editorial assessment and review synthesis

**Editor's summary and assessment**	This study presents long-read sequencing (Oxford Nanopore) and structural variant (SV) analysis, using activated T cells collected from 111 Japanese trios in the Tohoku Medical Megabank Biobank. This sample collection methodology is presented as a potential alternative to current biosamples used for generation of such genotype data (e.g. EBV-immortalised LCLs). The SV analysis focuses only on insertions and deletions, not more complex rearrangements, while the results seem largely in line with other large-scale studies of SVs using long reads. Several SVs with known phenotypic associations are observed in the data.<br><br>In our pre-review assessment, we thought that the use of the activated T cells was a potentially useful technical novelty, while the cohort of trios is a useful resource for the community. However, the overall conceptual and biological novelty seemed more limited, as the majority of the findings seem in line with the literature regarding long-read sequencing and SV analysis. Therefore, given the positive (technical and data) aspects of this submission, we thought that peer review was justified, most likely for consideration at *Communications Biology*.
**Editorial synthesis of reviewer reports**	The three reviewers have offered substantial and detailed reports, and acknowledge the potential that had been editorially noted at submission, noting that this work is of high quality and presents a useful resource for the field.<br><br>Each report raises a range of concerns, however, touching upon most aspects of this study; some concerns appear in multiple reports, suggesting they would be especially important to address. For example, the use of activated T cells was of interest, but a more comprehensive comparison to LCL data is needed; while the SV analysis was seen as offering limited novelty, and should be greatly expanded to improve on this.<br><br>While *Nature Genetics* and *Nature Communications* are unable to offer a revision, *Communications Biology* would be interested in considering a manuscript that (at a minimum) includes the following revisions:<br>1. Please evaluate any differences in insertions (as suggested by Reviewers 1-2)<br>2. Please carefully proofread for clarity and readability, the meaning/utility of "intact" DNA, and elaborate on potential advances presented over relevant studies, as outlined by Reviewers 2-3.<br>3. Please switch the data to hg38, as noted by Reviewers 1 and 3. For the sake of reproducibility, we would also ask that you deposit your sequencing data in a public database in advance of the next submission (for reviewer input).<br>4. If feasible, we would encourage you to include long-read sequencing data for a subset of LCLs, as suggested by Reviewer 1.

Editorial recommendation

Nature Genetics<br><br>Revision not invited	Due to the limited conceptual and biological novelty, *Nature Genetics* would not invite a revision.
Nature Communications<br><br>Revision not invited	Due to the limited conceptual and biological novelty, *Nature Communications* would also not invite a revision.
Communications Biology<br><br>Major revisions with extension of the work	As noted by the reviewers, analysis of insertions, further discussion of the importance of intact DNA and other relevant studies, use of a hg38 annotation would be especially important for further consideration.

Next steps

**Editorial recommendation:**	Our top recommendation is to revise and resubmit your manuscript to *Communications Biology.* We feel that the suggested comparison to LCL data would be especially important to demonstrate the value of using T cells, and the suggestions to improve the SV analysis *in silico* would also substantially add to the biological novelty of your study.
**Note**	As stated on the previous page, *Nature Genetics* and *Nature Communications* are not inviting a revision at this time. Please keep in mind that the journals will not be able to consider any appeals of their decision through Guided Open Access.

Revision

To follow our recommendation, please upload the revised manuscript files using **the link provided in the decision letter**. Should you need assistance with our manuscript tracking system, please contact Adam Lipkin, our Nature Portfolio Guided OA support specialist, at guidedOA@nature.com.

Revision checklist

- ☐ Cover letter, stating to which journal you are submitting
- ☐ Revised manuscript
- ☐ Point-by-point response to reviews
- ☐ Updated Reporting Summary and Editorial Policy Checklist
- ☐ Supplementary materials (if applicable)

Submission elsewhere

If you choose not to follow our recommendations, you can still take the reviewer reports with you.

Option 1: Transfer to another Nature Portfolio journal
Springer Nature provides authors with the ability to transfer a manuscript within the Nature Portfolio, without the author having to upload the manuscript data again. To use this service, **please follow the transfer link provided in the decision letter.** If no link was provided, please contact guidedOA@nature.com.

> *Note that any decision to opt in to In Review at the original journal is not sent to the receiving journal on transfer. You can opt in to In Review at receiving journals that support this service by choosing to modify your manuscript on transfer.*

Option 2: Portable Peer Review option for submission to a journal outside of Nature Portfolio
If you choose to submit your revised manuscript to a journal at another publisher, we can share the reviews with another journal outside of the Nature Portfolio if requested. You will need to request that the receiving journal office contacts us at guidedOA@nature.com. We have included editorial guidance below in the reviewer reports and open research evaluation to aid in revising the manuscript for publication elsewhere.

Annotated reviewer reports

The editors have included some additional comments on specific points raised by the reviewers below, to clarify requirements for publication in the recommended journal(s). However, please note that all points should be addressed in a revision, even if an editor has not specifically commented on them.

Reviewer #1 information

**Expertise**	genetics; long-read sequencing
**Editor's comments**	This reviewer has submitted a detailed report ranging over all aspects of your manuscript. Their requests seem largely reasonable and, we believe, would improve your study if fully addressed in a revision.

Reviewer #1 comments

**Section**	**Annotated Reviewer Comments**
**Remarks to the Author: Overall significance**	In recent years, long-read sequencing technologies have emerged as a superior method for the detection of structural variants (SVs) within genomes. In this manuscript "Construction of a trio-based structural variation panel utilizing activated lymphocytes and long-read sequencing technology" Otsuki and colleagues performed high coverage (~22X) nanopore sequencing on 111 Japanese trios using activated T lymphocytes as the source of genomic material. They identified a total of 68,571 SVs that are > 50 bp is length (34,341 deletions and 34,230 insertions), with 93.2% of them concordant with Mendelian inheritance. They compared the deletion calls to those from the Iceland population (deCODE dataset), provided a general functional annotation of identified SVs and a few examples of potentially clinically relevant deletions.<br><br>Please find below my specific comments:<br><br>One of the suggestions made by the authors for future studies is the use of activated T cells rather than EB virus-transformed B cells. There have been reports suggesting that B and T cell lymphocytes may demonstrate increased levels of genomic instability post VDJ-recombination. The authors should attempt to demonstrate that RAG-mediated or RSS-dependant transposition events during the T cell 'maturation' stages are indeed very infrequent (or even absent) effects *in vitro*, by directly comparing long-read sequence data generated from donor-matched lymphoblastoid (essentially B-cell) and T-cell samples. The authors are in a good place to ask this question as they are in possession of both the lymphoblastoid and activated T cell material for some of their samples (sequencing a few trios would suffice). This would be very useful both in terms of demonstrating that activated T cells are a superior (and safer) methodology, but also in ensuring that the results are not affected by

differences in somatic hypermutation related effects between B and T-cells. Given that a number of large population cohorts (e.g., the 1000 Genomes Project) are lymphoblastoid cell lines, this would be an excellent way to look into potential differences between B- and T-cells and support a shift in the field towards alternative sources of DNA.

> **If feasible (at least in a subset of samples), we think that a direct comparison of the T cell data to LCLs would substantially improve the impact of your study for the field, as suggested here.**

Comprehensive and accurate identification of SV from long-read data remains a challenge. The authors focused on the detection of only deletions and insertions but not other types of SVs (e.g., inversions) nor did they explain why other SVs were not included in the analyses. With the generated data they could attempt to identify at least a proportion of those. Then, for downstream analyses (e.g., the comparison to the deCODE dataset, and the Results section "SVs associated with clinical phenotypes"), the authors focused on deletions and ignored insertions. Including insertions and other types of SVs in the downstream analyses would be important and substantially strengthen the manuscript.

> **Expanding the analysis to evaluate insertions would be necessary for further consideration in *Communications Biology*.**

For the deletion and insertion detection, the authors only used one algorithm CuteSV and claimed its superiority (lines 317-318: "Nonetheless, we used CuteSV in this study, as this variant caller is assumed to be the most accurate available to date."). Such a statement should be supported by references. Best practices in the field incorporate multiple SV calling algorithms. Have the authors compared the CuteSV results with other widely-used SV calling algorithms, such as pbsv, sniffles and SVIM etc? Have they evaluated the performance of one algorithm vs combining multiple algorithms? The authors did not justify why they used only one algorithm and ideally this new method should be compared to existing methods to demonstrate its efficiency and accuracy, while the authors have not presented any such comparative analysis.

> **To ensure the quality of SV calls, at least one other algorithm should be used. At an absolute minimum, please justify why it was sufficient to solely rely on CuteSV.**

I wonder why the authors are still using the old hs37d5 (released in 2009) for their analysis when the human reference GRCh38 version has been widely used in most human genome studies since its release in 2013. The authors should re-analyse all the data using a more recent human reference (at some point even switching to T2T-CHM13 reference https://doi.org/10.1101/2021.05.26.445798). Additionally, if the authors mapped the sequence data to the GRCh38 reference, the results would be readily comparable to other published studies in the field without the need of liftover datasets from GRCh38 to hg19 or vice versa.

> **Please note that this issue is also raised by other reviewers.**

There is a substantial difference in the overall proportion of deletion and insertion variants identified in this study compared to previous studies (e.g., deCODE - https://doi.org/10.1038/s41588-021-00865-4, Ebert et al 2021 - DOI: 10.1126/science.abf7117). The current study identified a total of 68,571 deletion and insertion variants, with roughly 50-50 ratio between the categories, while this ratio is closer to 40-60 in both above-mentioned studies. Considering the total number of identified SVs, this is a substantial difference that would be important to elaborate on. As also mentioned by the authors, calling insertions accurately is more challenging than calling deletions, so perhaps a substantial number of insertion variants have remained unidentified in the current study indicating technical limitations (see the previous comments about SV calling algorithms and the use of hs37d5 as reference).

The authors have only compared their deletion calls to the deCODE dataset but not the insertion calls and no comparison has been performed with other recent publications or SV databases (e.g., DGV, gnomAD). This would be a very important addition.

Data availability: it is not clear whether the generated SV dataset is publicly available or not. At the Discussion section, lines 345-347, it says “The SVs identified in this study and their allele frequencies are publicly available at our website, the jMorp...”. At the Data availability section, lines 491-495, it says “The SV dataset will be available at the jMorp”. I was not able to find the SV dataset at the JMorp website. Can the authors make sure to submit the raw data and/or SV dataset before submission so that reviewers can assess the data and that the data will be available to the general scientific community after acceptance of the publication?

> **Please note that sequencing data must be made publicly available in an appropriate repository prior to acceptance. We would ask that you deposit your data prior to the next submission, so it can be assessed by the referees.**

The authors have not explained why the sex chromosomes have been excluded from their analyses.

The Results section “SVs associated with clinical phenotypes” could be improved by including examples of insertion variants and more detailed analysis of associations to phenotypes. Is there any phenotypic information available for the sequenced individuals that could be used to investigate associations with SVs? Admittedly, the sample size is small, but nevertheless some interesting examples could be provided.

Lines 265-267 “This observation supports the hypothesis that there are structure-variant hotspots in the human genome and that these peaks may correspond to these hotspots. In fact, the blue arrow position on chromosome 6 involves human leukocyte antigen (HLA) loci.” – did they look at the other “hotspot” regions in more detail? If not, then perhaps it would be interesting to do so.

According to Figure 5b, there are only 4 of such hotspots, so a more detailed analysis of these "hotspot" regions would be doable, and would definitely add value to the paper.

This point would be necessary for further consideration at *Communications Biology*.

It would be very interesting to add a more thorough analysis of SVs showing Mendelian inheritance errors (MIE) – what kind of SVs are they, the sequence composition, genomic location, how many are located in coding sequences, is it possible to separate biological from technical errors etc.

This point would be necessary for further consideration at *Communications Biology*.

Why did the authors estimate allele frequencies for 65,383 SVs, and not all 68,571 SVs?

It is not clear how the authors overcome the high error rate of nanopore reads. In line 160, they mentioned that they used a Phred score of 6 to filter reads but no further analyses were performed.

Line 163: the authors did not explain how well nanopore reads cover unique and repetitive regions (which are usually very difficult to assemble).

Line 204: "Because comparison of INSs is technically challenging, ….". The authors should elaborate why this is the case.

Lines 254-276: the authors could provide more details in this part, and could use a different plot to show the number of SVs on each chromosome to highlight how some chromosomes have more SVs compared to others even when the size of the chromosome is smaller.

Lines 272-274 "Thus, SVs located in intergenic regions were overrepresented and SVs in introns, exons, and CDSs were underrepresented (Fig. 5d). We also observed elevated rates of rare alleles in exons and CDSs (Fig. 5e).". Surely this is not unexpected and perhaps is worth comparing to the deCode or other published studies.

Minor comments:
Lines 70-72 - the statement requires a reference.

Line 157 - the authors could provide the exact number of males and females

Lines 190-195 - please add the size for different SVs.

Lines 266 - typo, "structure-variant" is not a term

	Line 270-271 "Of 68,571 SVs, 28,720 are located in intergenic regions; 38,524, 3,099, and 825 SVs overlap with introns, exons, and CDSs, respectively." Please add percentages to this sentence.<br><br>Line 283 - Please add the Table # for the supplementary Table<br><br>Line 443 – the degree symbol has been lost.<br><br>Line 588 and line 596 - reference 32 and 36 are duplicates.<br><br>Figure 2b and 2c – I would suggest adding the means to the plots as vertical lines as well.<br><br>Figure 4 b – is it minimum coverage that is plotted on the x-axis, or is it mean coverage?<br>There is no Figure 6f though it is referred to at least twice (line 299 and line 305).<br>Figure 7 – feels unnecessary – I would suggest to either move it to supplementary or remove entirely.<br><br>Supplementary Figure 1 – the colouring of deletions and insertions is potentially misleading and leaves an impression like in some regions only one or the other SV type is found. Unless this is the case I would suggest using a partially transparent colouring.<br><br>Supplementary Table. Protein coding genes overlapped with SVs – it would be useful to add the genomic location of these genes, size of identified SVs and the allele frequency/number of observations of overlapping SVs.

Reviewer #2 information

**Expertise**	genetics; long-read sequencing; bioinformatics
**Editor's comments**	This referee's report is focused on the area of their expertise, the bioinformatic analysis.

Reviewer #2 comments

Section	Annotated Reviewer Comments
**Remarks to the Author: Overall significance**	The authors present a study based on the Oxford Nanopore Technologies (ONT) long-read sequencing of 333 Japanese individuals from 111 parent-offspring trios. They designed a sample preparation protocol that enables high quality

	and high throughput DNA acquisition and were able to use the recommended ONT SV detection pipeline with good SV detection accuracy, shown by the Mendelian error rate estimates and genotype distribution. The results shown confirm the findings of previous cited work. While this work is a first example of a large-scale long-read sequencing for the Japanese population, it should focus on what further knowledge is gained from such a Japanese reference SV dataset from long-reads, combined with utilizing activated T lymphocytes.
**Remarks to the Author: Impact**	For this manuscript as is, *Communications Biology* is more suitable among the suggested ones. (i.e. *Nature Genetics*, *Nature Communications*, and *Communications Biology*). Although the data analysis results are currently confirmatory of previous long-read studies, if focus on a Japanese SV reference dataset is made, and what further knowledge can be obtained from its analysis it could increase the contribution of this study in the field. Otherwise, if the authors prefer to highlight the utilization of activated T lymphocytes for long-read sequencing on SV discovery, then the paper can benefit from focusing on sample preparation.
**Remarks to the Author: Strength of the claims**	My comments:<br><br>1) l. 26: enable better* characterization of SVs. (characterization of SVs have already been achieved to a degree using short read sequencing.)<br><br>2) l. 39-40: This sentence is unclear. Also, previous population scale short read and long read sequencing studies analyzing SVs already achieved that. The authors can focus what is achieved in this study on top of previously published literature.<br><br>3) l. 43: reword "genome medicine". Perhaps "genomic medicine"?<br><br>4) l. 46: rewrite: "genetic diversity and disease biology".<br><br>5) l. 51: I do not understand why filtering neutral variants is listed in this sentence. Neutral variants, i.e. variants that are neither beneficial nor detrimental, are not filtered out from a generic catalog of variants.<br><br>6) l. 65: connection to the next paragraph is broken.<br><br>7) l. 86: "has enabled new challenges" sounds strange. I suggest "resulted in (or created) new challenges"<br><br>8) l. 102: Other published strategies (also cited in this work) previously enabled effective construction of population-scale SV panel generation. I suggest using "allows for an effective construction ...".<br><br>9) l. 151-152: The aim presented is unclear.

10) l. 153: "111 parent-offspring trios".

11) l. 156-158: These sentences belong to another section.

12) l. 163: Was there a specific reason to use hg37d5 as opposed to a newer reference DNA such as hg38?

13) l. 156-166: The authors should also present the sequencing error rates (insertion, deletion, mismatch, and total).

For the sake of reproducibility, this point would be necessary for further consideration at *Communications Biology*.

14) l. 175 The authors should point to the methods for SV merging.

15) l. 176 Why does it make sense to observe a balanced number of DELs and INSs. Comparison to other large scale long-read SV works needs to be provided here.

16) l. 181: I do not see how the data presented supports this claim.

17) l. 184: What does it mean "we extracted SVs observed" here? Did you simply use the discovered SVs that are members of a merged SV, or did you genotyped the merged SVs in the individuals?

18) l. 191-195: The observation made here requires a proper statistical analysis with hypothesis testing. On the one hand, it is natural to expect large SVs to be more disruptive thus to be rare. On the other hand, false positive SVs may also lead to such findings, and one would expect false positive SVs to tend to appear more as singletons.

For the sake of reproducibility, this point would be necessary for further consideration at *Communications Biology*.

19) l. 198: Why not insertions as well as deletions?

20) l. 201: the deCODE dataset does report the frequency of the SVs. Please find it in Supplementary Data 2.

21) l. 204: There exists a series of SV comparison approaches, used for comparing insertions as well, in the previous works cited in this manuscript. The authors could simply pick one and apply it for insertions as well. Otherwise, the lack of any comparison on roughly half of the merged SV set results in an incomplete analysis.

This point was also raised by Reviewer #1.

22) l. 206: This sentence needs a pointer to the methods for SV comparison.

	23) l. 197-212: I do not think it is useful to compare rare or singleton SVs to the deCODE dataset. I would suggest comparing common SVs. Also this sections, as is, constitutes more of a quality-control rather than providing insight into the differences in ethnic distributions of SVs, does very minimally sheds light on the ethnic diversity of SVs.<br><br>24) l. 214-251: This entire section is basically the distribution of SV genotypes in parent-offspring trios, as presented in Fig 4c. This section could be drastically shortened, mainly referring to the figure and mention that the behaviour is comparable to the decode study.<br>**We would leave this point to the discretion of the authors.**<br><br>25) l. 265-268: It is well established that SVs are over-represented in centromeric and subtelomeric regions.<br><br>26) l. 269-276: The section that discusses the over and under-representation of SVs within different regions, i.e. intergenic, intronic, exonic, ... etc. requires p-values rejecting the null hypothesis of having no over or under representation.<br><br>27) l. 302: A 32kb deletion in LCE3B also is reported in the decode study, associated with psoriasis, which should be cited in this section.<br><br>28) l. 317: As an alternative, the authors can use a long read SV genotyper such as LRcaller, which could provide a better estimate of allele frequency, as opposed to simply using the genotypes from an SV discovery tool.<br><br>29) l. 327: The discussion section can be substantially shortened.
**Remarks to the Author: Reproducibility**	I mentioned the required further statistical analyses in the section above.<br><br>The required parameters for the software being run are provided.

Reviewer #3 information

**Expertise**	genomics; long-read sequencing
**Editor’s comments**	This reviewer's report is focused on the technical aspects of the sample collection and basic data quality. They think that your manuscript makes some claims that are, in their view, unsubstantiated and may cause confusion in the field; it would be important to ensure that these have been fully addressed in a revision.

Reviewer #3 comments

Section	Annotated Reviewer Comments
**Remarks to the Author: Overall significance**	Firstly my apologies to the authors for a long turnaround on this review.<br><br>The paper by Otsuki and colleagues describes the use of nanopore sequencing to investigate structural variation in a sample of 111 trios from the Japanese population. To address the difficulty of obtaining long DNA they develop an approach to use activated T-cells which are cultured from a BioBank, demonstrating a useful source of material where long DNA has not been acquired previously. The paper goes on to demonstrate the utility of promethION sequencing to ask questions about the presence of SVs throughout the genome and investigates the concordance (or otherwise) of these events.<br><br>Overall the work is carried out to a high standard and will provide a useful resource for the future. I hope the SV database will be accessible and straightforward to access with appropriate permissions.<br><br>A major focus of the paper is the use of T-cells to provide DNA. Whilst I think this is an interesting and novel approach I am not sure it is an absolute requirement for sequencing projects of this nature. A comparison with DNA extracted for the same samples for short read sequencing but run on a long read platform would reveal the benefits of the T-cell method. It is important to not give the impression that read N50s of the type generated here are absolutely required for the level of SV detection observed unless this is actually the case. However, the use of the T-cells is an innovative approach.
**Remarks to the Author: Impact**	The paper provides a useful resource for long read technologies and their application but I am concerned that it suggests higher molecular weight DNA is required for a study of this nature than is actually required. Thus the paper in its current form may influence thinking in the field but by reinforcing unsupported views.
**Remarks to the Author: Strength of the claims**	1. The authors contend that a limitation on long read sequencing is the requirement for "large amounts of intact DNA". In the following sentence they argue that "In general, the molecular weight of the genomic DNA used for library preparation and its purity substantially affect variant detection, particularly for large SVs, and data yield.". They support this argument with reference to a review from 2019. It would be helpful for the authors to precisely quantify what they mean by a large amount of intact DNA. We routinely run sequencing from as little as 1ug of starting material and the review is not specific in this regard - other users have reported good performance from 300 ng or less. Furthermore, the review doesn't really

	address the argument the authors make that molecular weight and purity substantially affect variant detection. If the authors are simply distinguishing between "short" and "long" reads then I agree that read length matters - but it is important to be clear on the distinctions. The paper from Beyter et al (long read sequencing from Iceland) has a significantly lower N50 distribution than the work presented here and yet they are also able to analyse SVs.<br>**Please qualify the necessity of "intact" DNA, as outlined above.**<br><br>2. The authors have a lot of data on the performance of their experiments but it isn't presented. A scatter plot of N50 against yield as well as purity vs yield would support the claims made. In addition, some measure of how the ability to detect SVs varies by read length would be ideal. We have had no problem in generating informative libraries for SV calling from DNA extracted for short read projects. Our read N50s have been in the range of 15-20 kb (similar to that from Beytar et al). I think the authors should demonstrate a link if it exists between the ability to SV call at N50s of 15 kb to 40 kb.<br>**Reviewer #2 also hinted at the need to disclose other parameters of experiment performance (point 13).**<br><br>3. Pg 7 - lines 135-137 - the authors assert that there is an inverse correlation between library yield and library molecule length (citing the same review). Again, this is a complex statement. Users can obtain high yields (100Gb) on samples prepared with the Nanopore ULK-001 kit for example. Again, the paper contains data that would allow that assessment to be made (plot N50 against yield over many experiments).<br><br>4. Pg 6. Line 110-11 - I am not sure on the statement "data yield per cost varies significantly dependent on the input.". Yield can be dependent on the quality of sample input, but this could be quantified in some way<br><br>5. Pg 7 - line 140-141. This sentence is not clear. Are the yields for reads greater than 20Kb? "More than 20kb of N50" is unclear. Figure 1e doesn't show anything about yield, rather it is showing read length against quality with a heat map of read count. Converting this to base pairs is somewhat complicated.<br><br>6. Pg 10 - lines 230-232 - can the authors elaborate on which technical improvements they think have caused the change?
**Remarks to the Author: Reproducibility**	1. Pg 12 - lines 259 - The authors show that SVs occur in close proximity to gaps in the reference and imply this is an artefact - to address this they simply omit these regions from their analysis. This led me to ask which reference they used. Close inspection reveals the authors used the hs37d5 reference based on the 1000 genomes project but not a later more complete assembly such as hg38 or a more recent telomere-to-telomere assembly. Obviously changing the entire study to a new reference is significant, but I wonder why the authors chose the

	earlier assembly base? They resolve the ambiguity of SVs in close proximity to gaps by removal from the analysis (if I understand correctly) but it would be interesting to determine if mapping to more contiguous reference resolved this issue.<br><br>2. Pg 20 - line 456 - I think the authors are referencing a mean Phred score > 6 - and so this should be described as a mean quality score? (i.e Nanopore "Pass" reads?).<br><br>3. Figure 1e - I'm not convinced this panel clearly presents the statements the authors make about it in the text (see 5).<br><br>4. Figure 2 - a scatter plot of N50 vs yield would be interesting (but should also include some reference to pore count on the flowcell).

Open research evaluation

Data availability
**Data Availability Statement**
Thank you for including a Data Availability statement. However, we noted that you have only indicated that data are available upon request. These statements are strongly discouraged by the research community as they do not make it clear which data are available and under what conditions. The data availability statement must make the conditions of access to the data underlying the study transparent to readers. Please explicitly state if and how readers may access the individual datasets reported in the study, including any unique identifiers or URLs and any restrictions to access that apply.<br><br>In addition, Nature Portfolio policies include a strong preference for research data to be archived in public repositories. For data types without specific repositories, we recommend that data are deposited in a generalist repository such as figshare or Dryad. More information about our data availability policy can be found here:<br>https://www.nature.com/nature-portfolio/editorial-policies/reporting-standards#availability-of-data<br><br>See here for more information about formatting your Data Availability Statement:<br>http://www.springernature.com/gp/authors/research-data-policy/data-availability-statements/12330880
This journal strongly supports public availability of data and custom code associated with the paper in a persistent repository where they can be freely and enduringly accessed or as a supplementary data file when no appropriate repository is available. If data and code can only be shared on request, please explain why in your data Availability Statement, and also in the correspondence with your editor. For more information, please refer to<br>https://www.nature.com/nature-research/editorial-policies/reporting-standards#availability-of-data
Please ensure that datasets deposited in public repositories are now publicly accessible, and that accession codes or DOI are provided in the "Data Availability" section. As long as these datasets are not public, we cannot proceed with the acceptance of your paper. For data that have been obtained from publicly available sources, please provide a URL and the specific data product name in the data availability statement. Data with a DOI should be further cited in the methods reference section.
**Mandatory data deposition**

DNA sequence data must be made publicly available in an approved repository as a prerequisite for publication in a Nature Portfolio journal. Deposition of these data is also best practice for publication in any venue. Accession numbers must be provided in the paper. Examples of appropriate public repositories are listed below:

GenBank
Sequence Read Archive (WGS or WES data)
The European Nucleotide Archive (ENA)

More information on mandatory data deposition policies at the Nature Portfolio can be found at http://www.nature.com/authors/policies/availability.html#data

Please visit https://www.springernature.com/gp/authors/research-data-policy/repositories/12327124 for a list of approved repositories for each mandatory data type.

Data publishing recommendations

You may also be interested in publishing a Data Descriptor with Scientific Data to improve the usability of your data set. See the journal website for details: https://www.nature.com/sdata/

For more information about data publishing at Springer Nature, please see https://www.springernature.com/gp/authors/research-data/research-data-publishing

Data citation

Please cite (within the main reference list) any datasets stored in external repositories that are mentioned within their manuscript. For previously published datasets, we ask that you cite both the related research article(s) and the datasets themselves. For more information on how to cite datasets in submitted manuscripts, please see our data availability statements and data citations policy: https://www.nature.com/documents/nr-data-availability-statements-data-citations.pdf

Citing and referencing data in publications supports reproducible research, by increasing the transparency and provenance tracking of data generated or analysed during research. Citing data formally in reference lists also helps facilitate the tracking of data reuse and may help assign credit for individuals' contributions to research. A number of Springer Nature imprints are signatories of the Joint Declaration on Data Citation Principles, which stress the importance of data resources in scientific communication.

Code availability and citation

Please include a statement under the heading "Code Availability", indicating whether and how the custom code/software reported in your study can be accessed, including any restrictions to access. This section should also include information on the versions of any software used, if relevant, and any specific variables or parameters used to generate, test, or process the current dataset. Code availability statements should be provided as a separate section after the Data Availability section.

Upon publication, Nature Portfolio journals consider it best practice to release custom computer code in a way that allows readers to repeat the published results. Code should be deposited in a DOI-minting repository such as Zenodo, Gigantum or Code Ocean and cited in the reference list following the guidelines described in our policy pages (see link below). Authors are encouraged to manage subsequent code versions and to use a license approved by the open source initiative. Full details about how the code can be accessed and any restrictions must be described in the Code Availability statement.

See here for more information about our code availability policies:
https://www.nature.com/nature-portfolio/editorial-policies/reporting-standards#availability-of-computer-code

We also provide a Code and Software submission checklist that you may find useful:
https://www.nature.com/documents/nr-software-policy.pdf

Please note: because of advanced features used in this form, you must use Adobe Reader to open the documents and fill it out.

Ethics

Because your study includes human participants, confirmation that all relevant ethical regulations were followed is needed, and that informed consent was obtained. This must be stated in the Methods section, including the name of the board and institution that approved the study protocol.

Reporting & reproducibility

Nature Portfolio journals allow unlimited space for Methods. The Methods must contain sufficient detail such that the work could be repeated. It is preferable that all key methods be included in the main manuscript, rather than in the Supplementary Information. Please avoid use of “as described previously” or similar, and instead detail the specific methods used with appropriate attribution.

Please state in the legends how many times each experiment was repeated independently with similar results. This is needed for all experiments, but is particularly important wherever results from representative experiments (such as micrographs) are shown. If space in the legends is limiting, this information can be included in a section titled “Statistics and Reproducibility” in the methods section.

Data presentation: Please ensure that data presented in a plot, chart or other visual representation format shows data distribution clearly (e.g. dot plots, box-and-whisker plots). When using bar charts, please overlay the corresponding data points (as dot plots) whenever possible and always for n ≤ 10. (Please see the following editorial for the rationale behind this request and an example https://www.nature.com/articles/s41551-017-0079).
Statistics: Wherever statistics have been derived (e.g. error bars, box plots, statistical significance) the legend needs to provide and define the n number (i.e. the sample size used to derive statistics) as a precise value (not a range), using the wording “n=X biologically independent samples/animals/cells/independent experiments/n= X cells examined over Y independent experiments” etc. as applicable.
Legends requiring revision:<br>1. Please note that this information is missing in the legends of figures 3d; 4a; 5d.
Statistics such as error bars, significance and p values cannot be derived from n<3 and must be removed from all such cases.
We strongly discourage deriving statistics from technical replicates, unless there is a clear scientific justification for why providing this information is important. Conflating technical and biological variability, e.g., by pooling technically replicated samples across independent experiments is strongly discouraged. (For examples of expected description of statistics in figure legends, please see the following https://www.nature.com/articles/s41467-019-11636-5 or https://www.nature.com/articles/s41467-019-11510-4).
All error bars need to be defined in the legends (e.g. SD, SEM) together with a measure of centre (e.g. mean, median). For example, the legends should state something along the lines of “Data are presented as mean values +/- SEM” as appropriate.<br>All box plots need to be defined in the legends in terms of minima, maxima, centre, bounds of box and whiskers and percentile.
Legends requiring revision:<br>1. Please note that the error bars need to be defined in the legend of figure 5d.<br>2. Please note that the measure of centre for the error bars needs to be defined in the legends of figures 5a, c.<br>3. Please note that the box plots need to be defined in terms of minima, maxima, centre, bounds of box and whiskers and percentile in the legend of figure 3d.
The figure legends must indicate the statistical test used. Where appropriate, please indicate in the figure legends whether the statistical tests were one-sided or two-sided and whether adjustments were made for multiple comparisons.
**Other notes**

We have included as an attachment to the decision letter a version of your Reporting Summary with a few notes. This is mainly for your information, but we hope it is helpful when preparing your revised manuscript. If you decide to resubmit the manuscript for further consideration, please be sure to include an updated Reporting Summary.

Revise (with EAR)

December 7th, 2021

Dear **Dr. Yamamoto,**

Thank you again for choosing to submit your manuscript using the Guided Open Access pilot at the Nature Portfolio. As part of this process, our editorial team has considered your paper for three of our journals with strong interest in publishing in your field: **<i>Nature Genetics, Nature Communications</i>, and <i>Communications Biology</i>.**

Your manuscript entitled "**Construction of a trio-based structural variation panel utilizing activated T lymphocytes and long-read sequencing technology**" has now been reviewed by **3** experts in **computational biology, genomics, and long-read sequencing**, whose comments are included below and in the attached Editorial Assessment Report. As part of the Guided Open Access pilot, editors from all 3 journals have discussed the reviewer reports and the manuscript's suitability for our journals. After careful evaluation, our editorial recommendation is to revise the manuscript and submit back through the Guided Open Access submission portal for consideration at **<i>Communications Biology</i>** using the link provided below. Provided the revisions satisfy all technical and editorial concerns, **<i>Communications Biology</i> is** very interested in publishing your manuscript. Please see details in the attached Editorial Assessment Report.

In brief, analysis of insertions, further discussion of the importance of intact DNA and other relevant studies, use of a hg38 annotation would be especially important for further consideration at **<i>Communications Biology</i>.**

Please note that the Editorial Assessment Report is a standalone document that contains an editorial evaluation, recommendation and portable peer advice to help you navigate and interpret the reviewers' reports. It also provides guidance for adhering to best practice with regard to transparency and reproducibility, for example on the issue of sharing data. We have also included information about data accessibility and reproducibility, which we hope you find useful.

HOW TO SUBMIT
Once you are ready to submit a revised version of your manuscript, please use the link below to submit the following items as separate documents:
-- Revised manuscript. Please show all changes in the manuscript text file with tracked changes or colour highlighting
-- Any supplementary files.
-- Point-by-point response to the reviewers' comments, reproduced verbatim. If you are unable to address specific reviewer requests or find any points invalid, please explain why.
-- Cover letter to the editor, stating the journal for which you have revised.

[SUBMISSION LINK]

Note: This URL links to your confidential home page and associated information about manuscripts you may have submitted, or that you are reviewing for us. If you wish to forward this email to co-authors, please delete the link to your homepage.

Should you have any questions about the recommended journals or would like advice on the revisions, you can contact me directly and I will be happy to assist. We look forward to receiving the revised version of your manuscript.

Yours sincerely,
[EDITOR]
--

[Editor name]
Editor
Guided Open Access

On behalf of the Guided OA editorial team
