# Supplementary material for: Construction of a trio-based structural variation panel utilizing activated T lymphocytes and long-read sequencing technology
Source: Commun Biol. 2022 Sep 20;5:991. doi: 10.1038/s42003-022-03953-1 (PMC9489684; doi:10.1038/s42003-022-03953-1)
Supplement: Supplementary file 4 — Description of Additional Supplementary Files [file 42003_2022_3953_MOESM4_ESM.pdf]

Description of Additional Supplementary Files

File Name: Supplementary Data 1

Description: Sequencing statistics for individual runs.

File Name: Supplementary Data 2

Description: List of protein coding genes overlapped with SVs.

File Name: Supplementary Data 3

Description: The numerical source data behind the figures.

File Name: Supplementary Software 1

Description: The in-house scripts (mendelian-check.py and gene-enrichment.py).

File Name: Supplementary Software 2:

Description: The codes behind the figures.
