# Supplementary material for: Construction of a trio-based structural variation panel utilizing activated T lymphocytes and long-read sequencing technology
Source: Commun Biol. 2022 Sep 20;5:991. doi: 10.1038/s42003-022-03953-1 (PMC9489684; doi:10.1038/s42003-022-03953-1)
Supplement: Supplementary file 9 — Supplementary Data 3 [file 42003_2022_3953_MOESM9_ESM.zip › SuppData3/fig3-f.pdf]

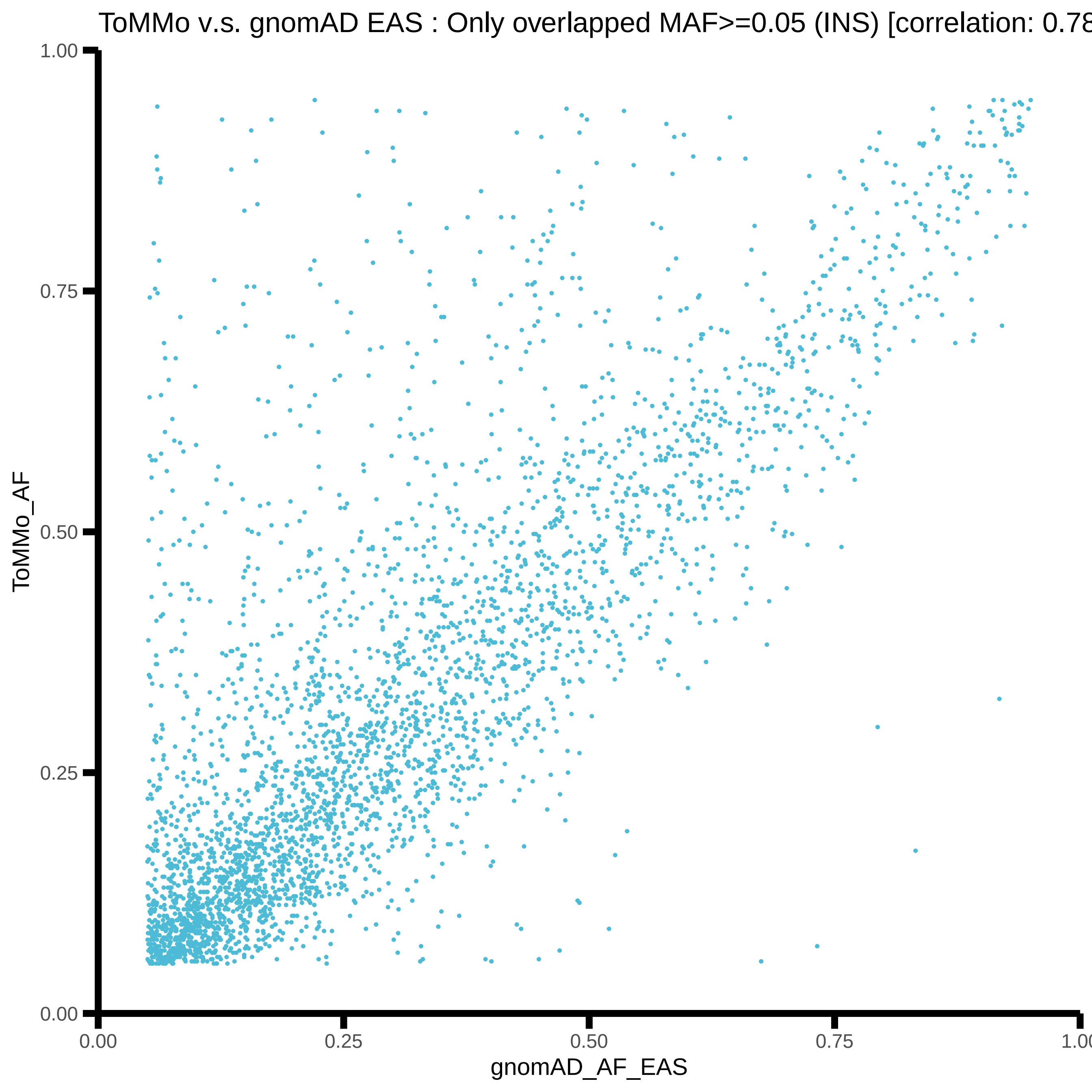
ToMMo v.s. gnomAD EAS : Only overlapped MAF>=0.05 (INS) [correlation: 0.78
ToMMo_AF
1.00
0.75
0.50
0.25
0.00
0.00
0.25
0.50
0.75
1.00
gnomAD_AF_EAS

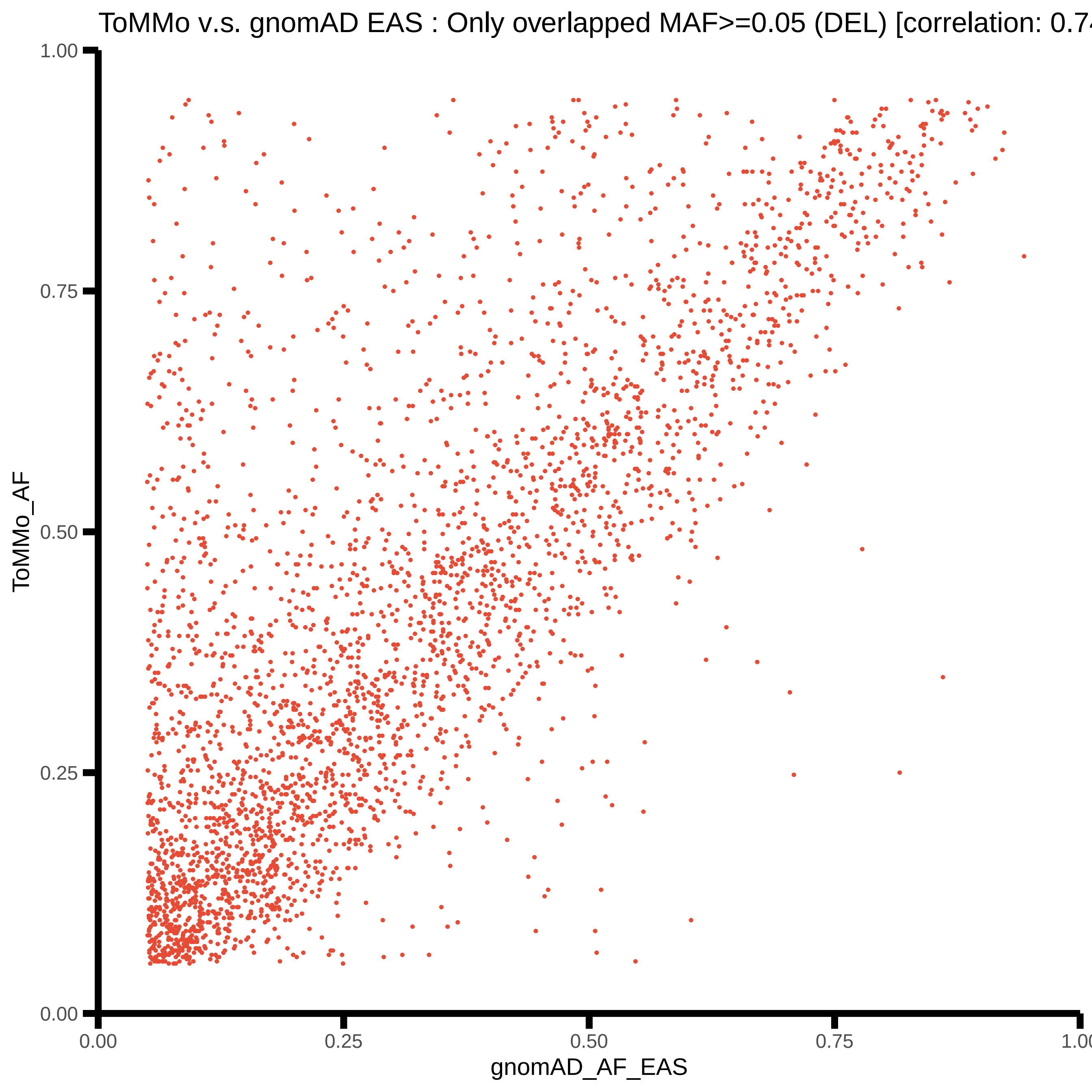

ToMMo v.s. gnomAD EAS : Only overlapped MAF>=0.05 (DEL) [correlation: 0.7
ToMMo_AF
gnomAD_AF_EAS
0.00
0.25
0.50
0.75
1.00
0.00
0.25
0.50
0.75
1.00
