# Supplementary figures and images for: Construction of a trio-based structural variation panel utilizing activated T lymphocytes and long-read sequencing technology
Source: Commun Biol. 2022 Sep 20;5:991. doi: 10.1038/s42003-022-03953-1 (PMC9489684; doi:10.1038/s42003-022-03953-1)

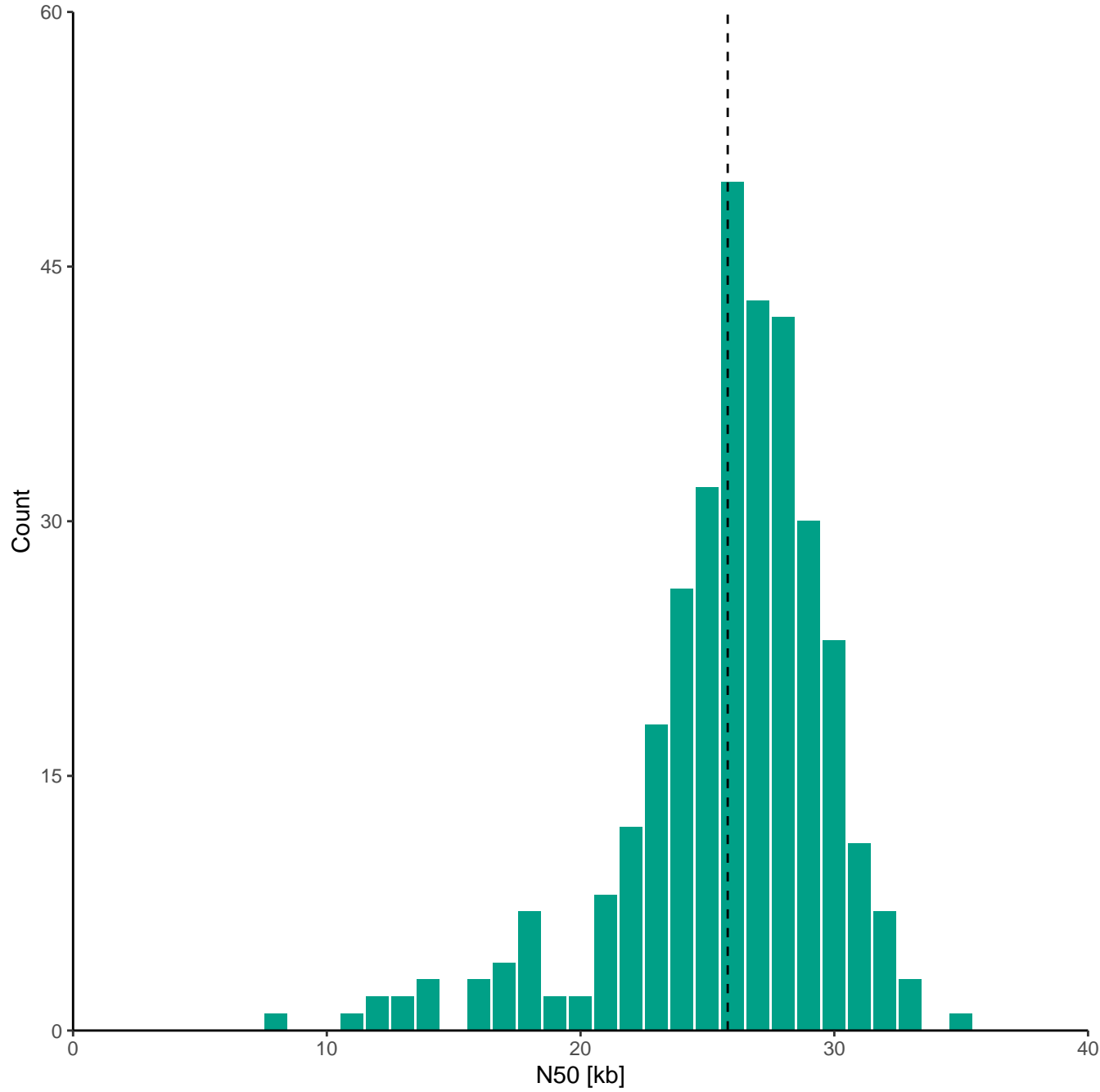

Supplement: Supplementary file 9 — Supplementary Data 3 [file 42003_2022_3953_MOESM9_ESM.zip › SuppData3/fig2-b.pdf]

TYPE DEL INS

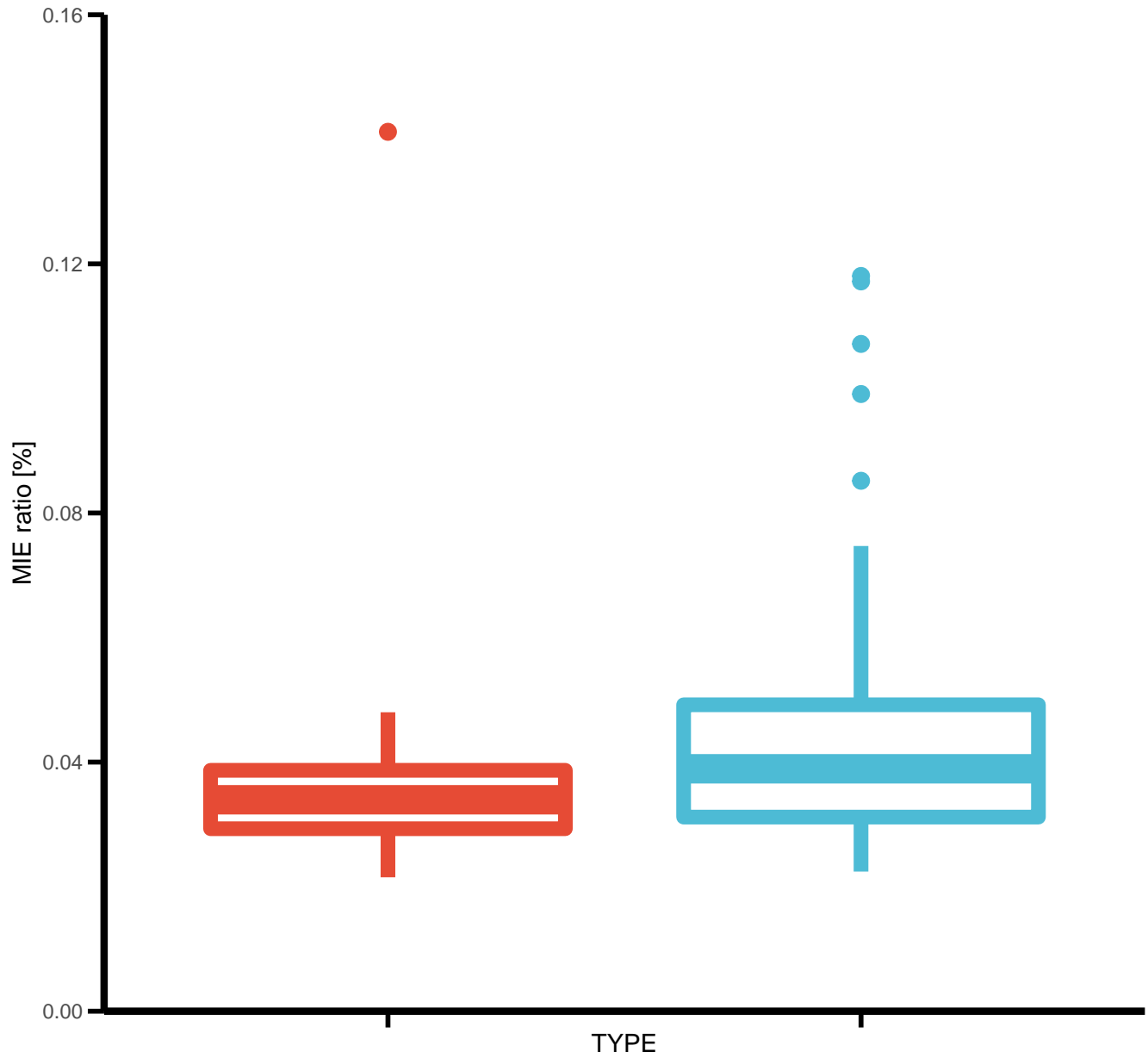

Supplement: Supplementary file 9 — Supplementary Data 3 [file 42003_2022_3953_MOESM9_ESM.zip › SuppData3/fig4-a.pdf]

name del.count ins.count

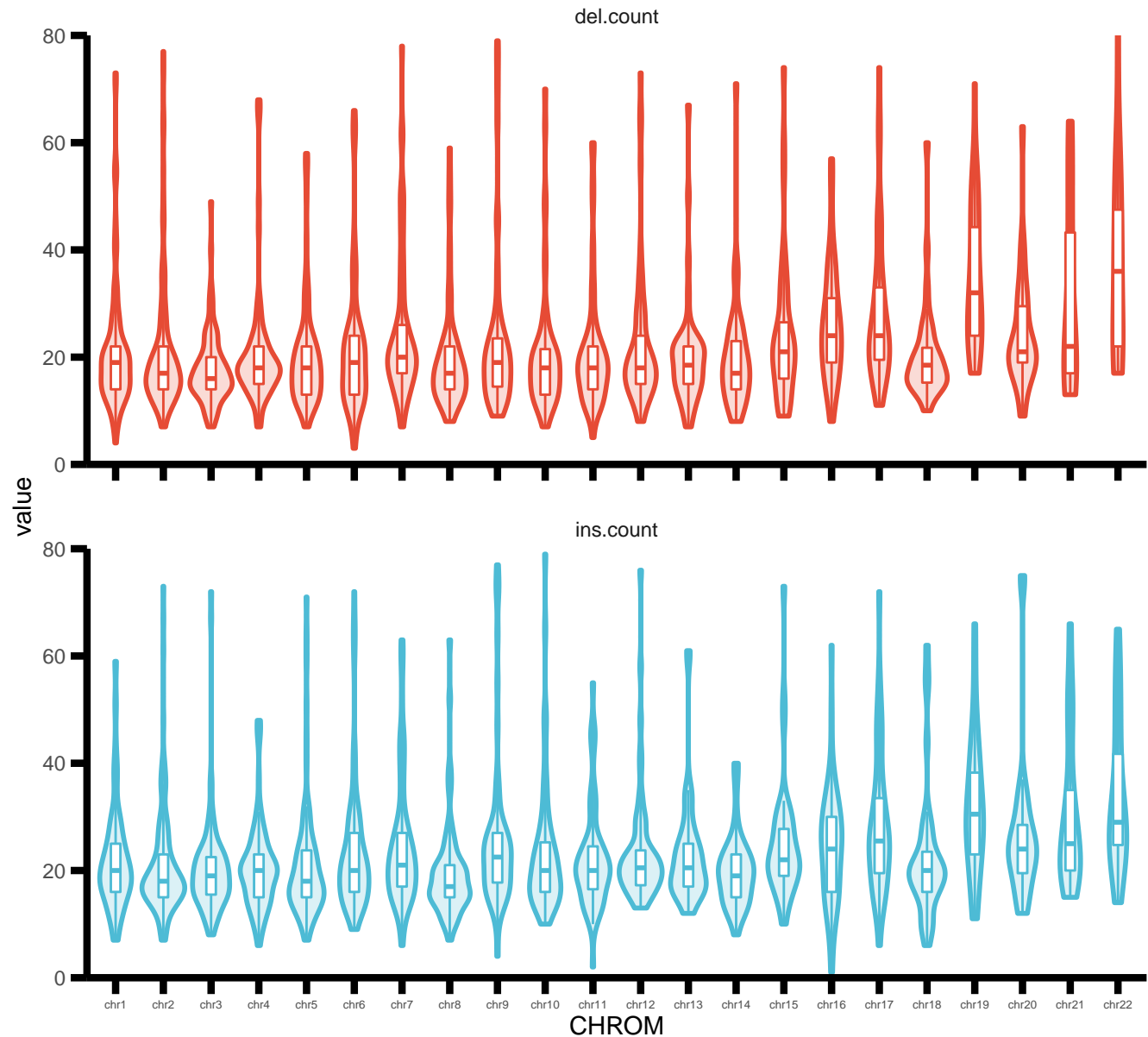

Supplement: Supplementary file 9 — Supplementary Data 3 [file 42003_2022_3953_MOESM9_ESM.zip › SuppData3/fig5-b.pdf]

SVTYPE DEL INS

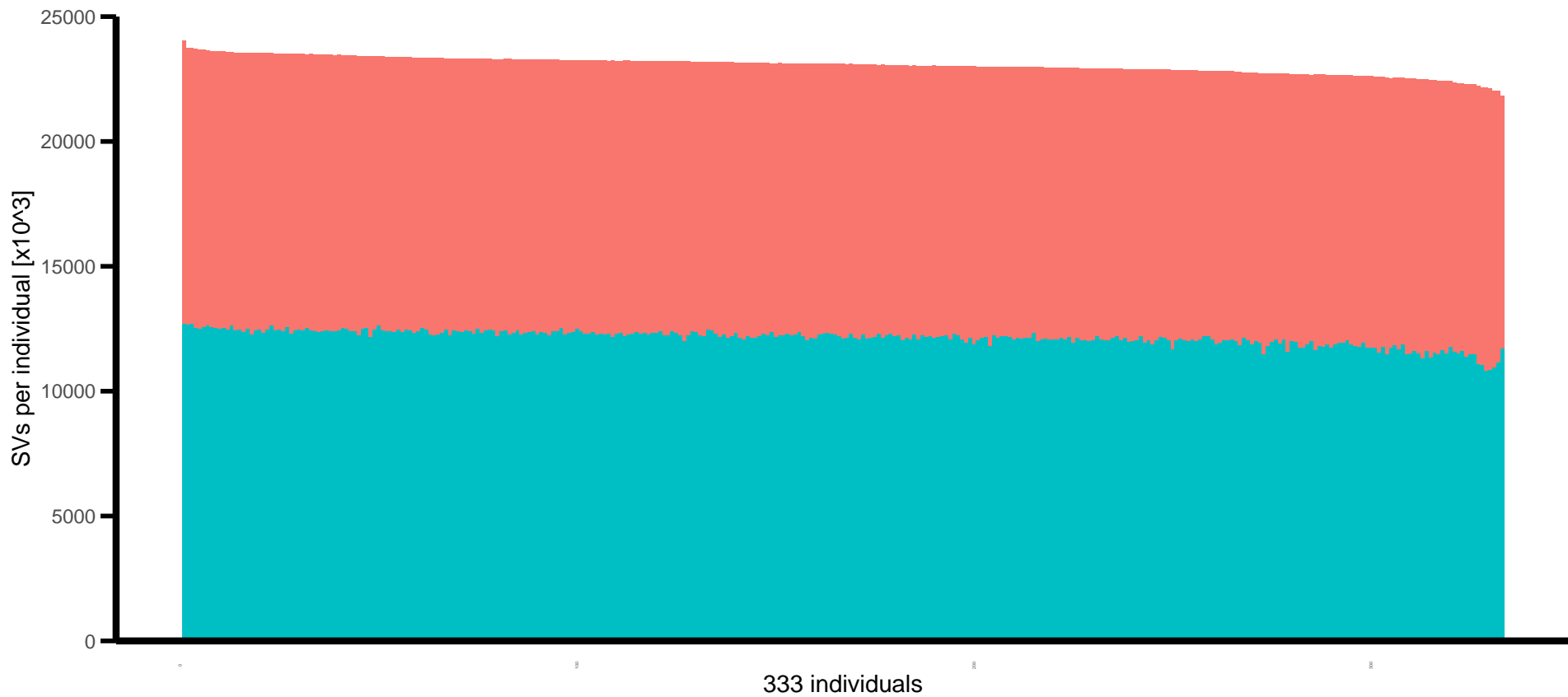

Supplement: Supplementary file 9 — Supplementary Data 3 [file 42003_2022_3953_MOESM9_ESM.zip › SuppData3/fig2-d.pdf]

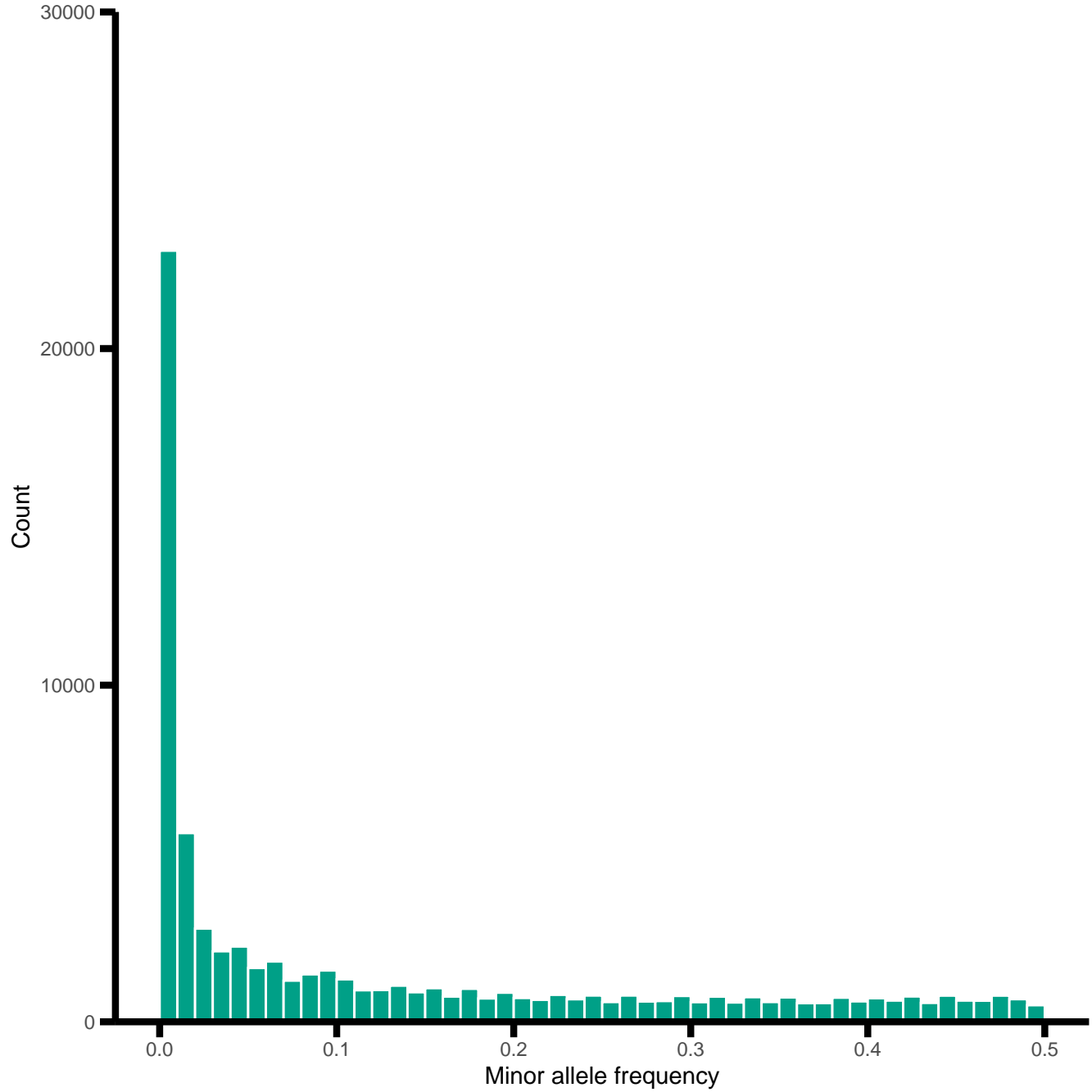

Supplement: Supplementary file 9 — Supplementary Data 3 [file 42003_2022_3953_MOESM9_ESM.zip › SuppData3/fig3-a.pdf]

mendelian\_autosome

Child 11 01 00

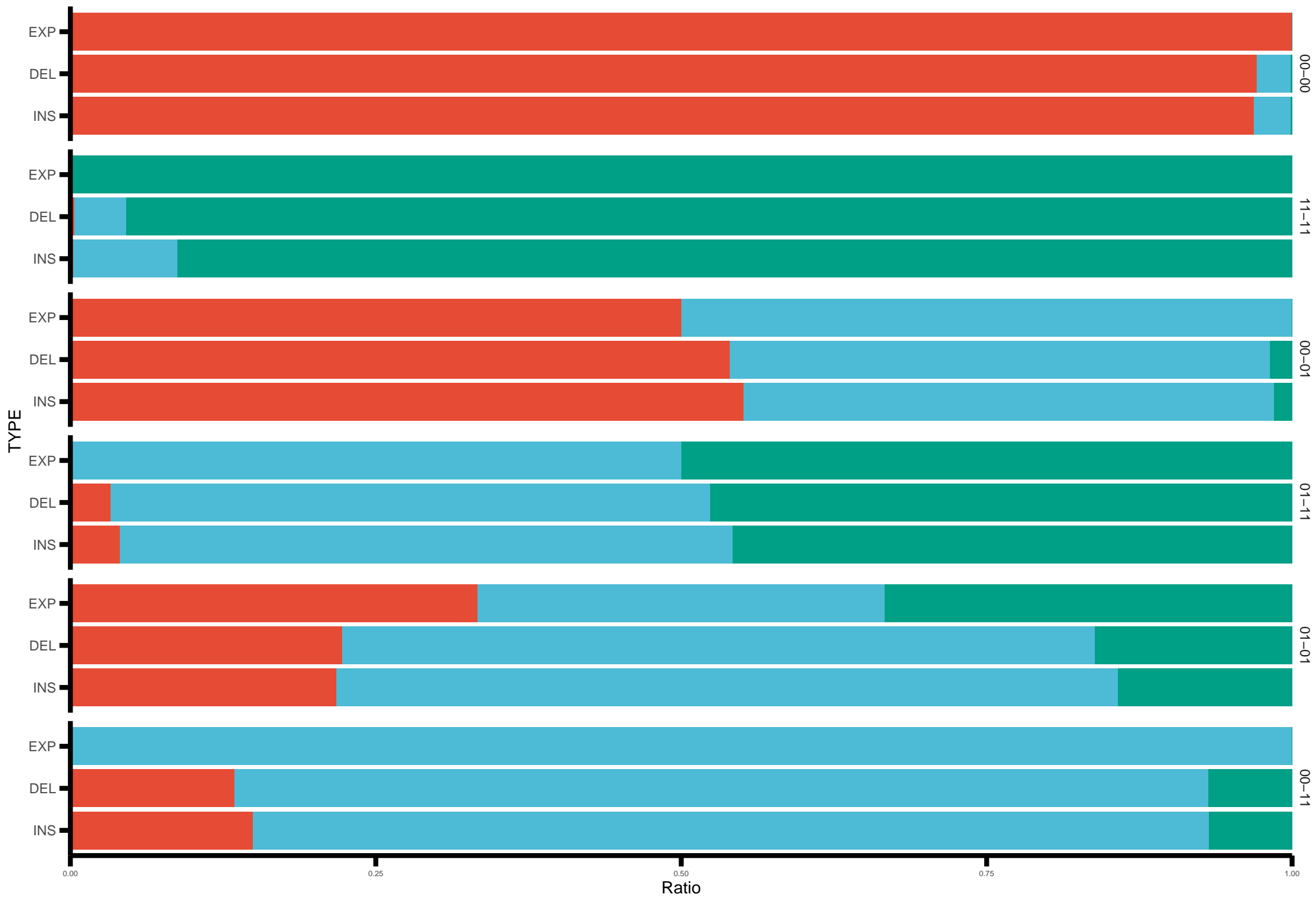

Supplement: Supplementary file 9 — Supplementary Data 3 [file 42003_2022_3953_MOESM9_ESM.zip › SuppData3/fig4-c.pdf]

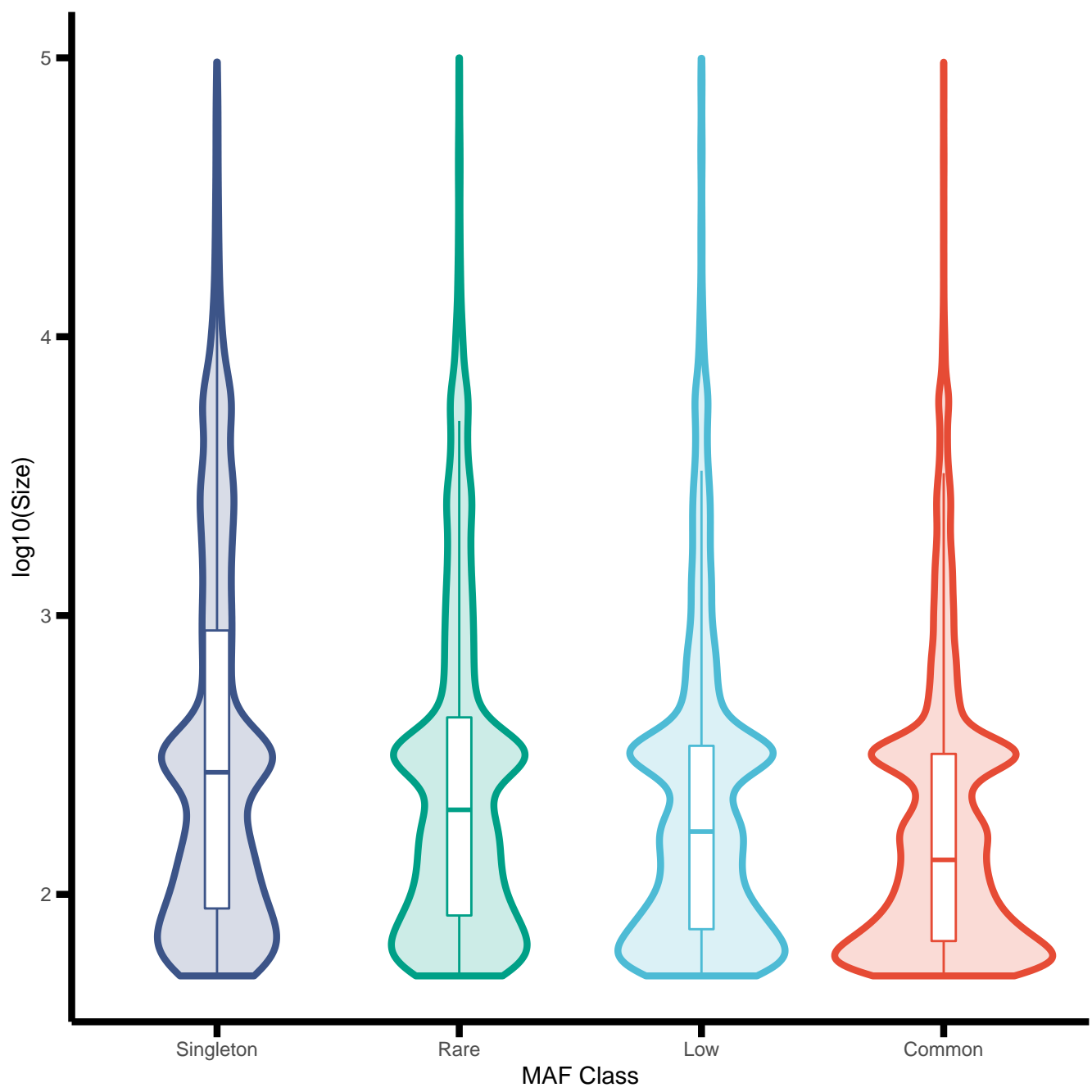

Supplement: Supplementary file 9 — Supplementary Data 3 [file 42003_2022_3953_MOESM9_ESM.zip › SuppData3/fig3-b.pdf]

MAF.CLASS    Singleton    Rare    Low    Common

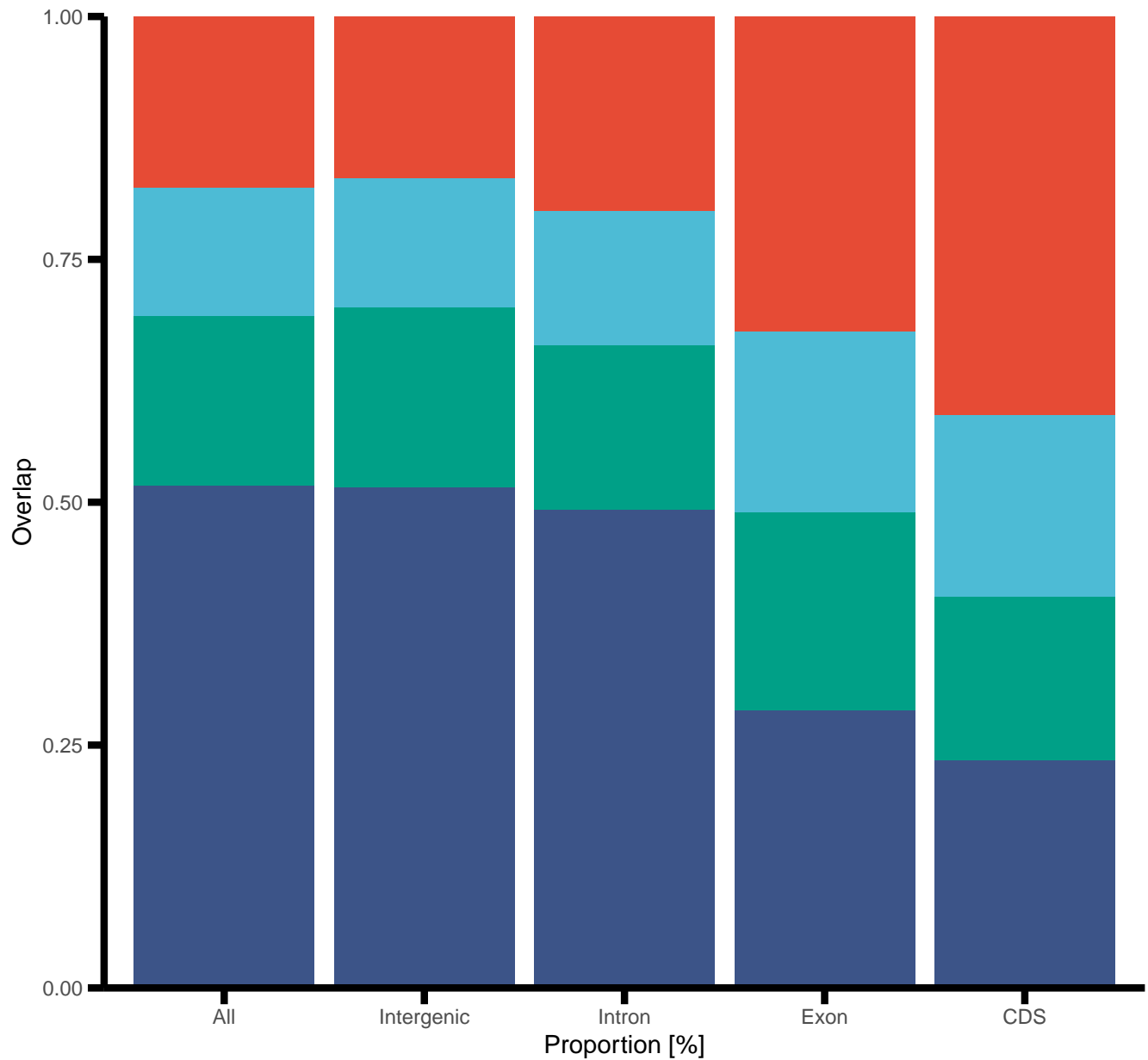

Supplement: Supplementary file 9 — Supplementary Data 3 [file 42003_2022_3953_MOESM9_ESM.zip › SuppData3/fig5-e.pdf]

Position v.s. SV Type

INFO.SVTYPE DEL INS

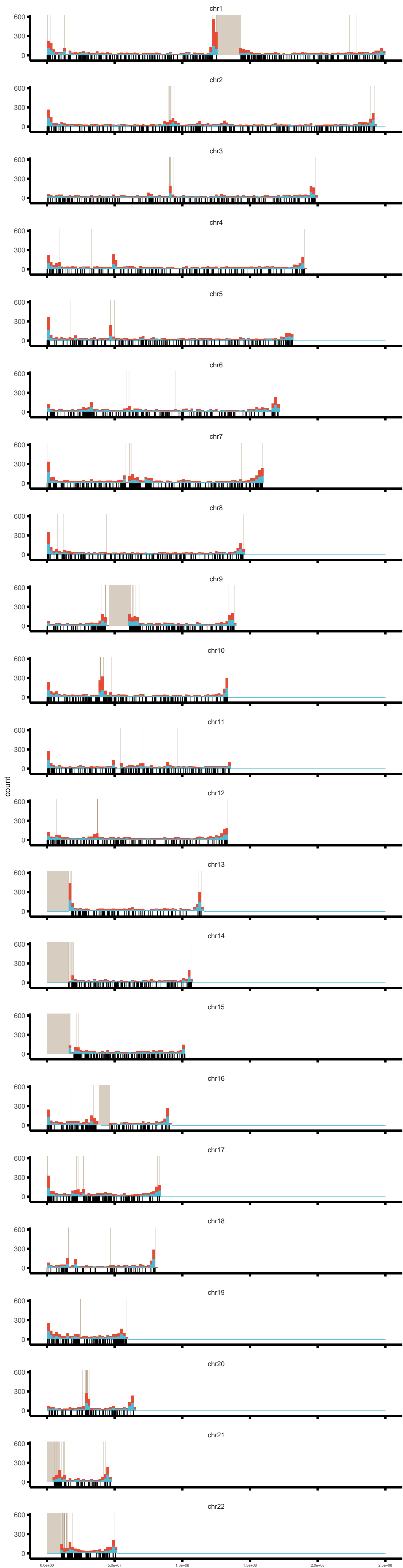

Supplement: Supplementary file 9 — Supplementary Data 3 [file 42003_2022_3953_MOESM9_ESM.zip › SuppData3/fig5-c.pdf]

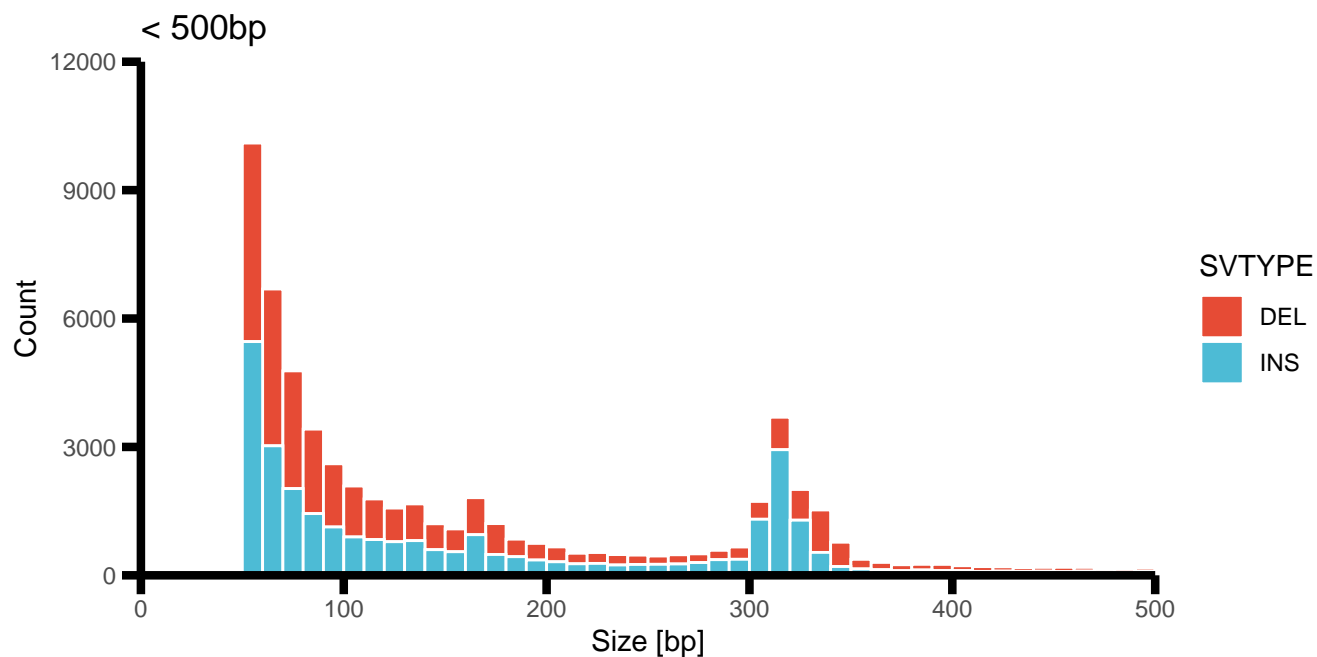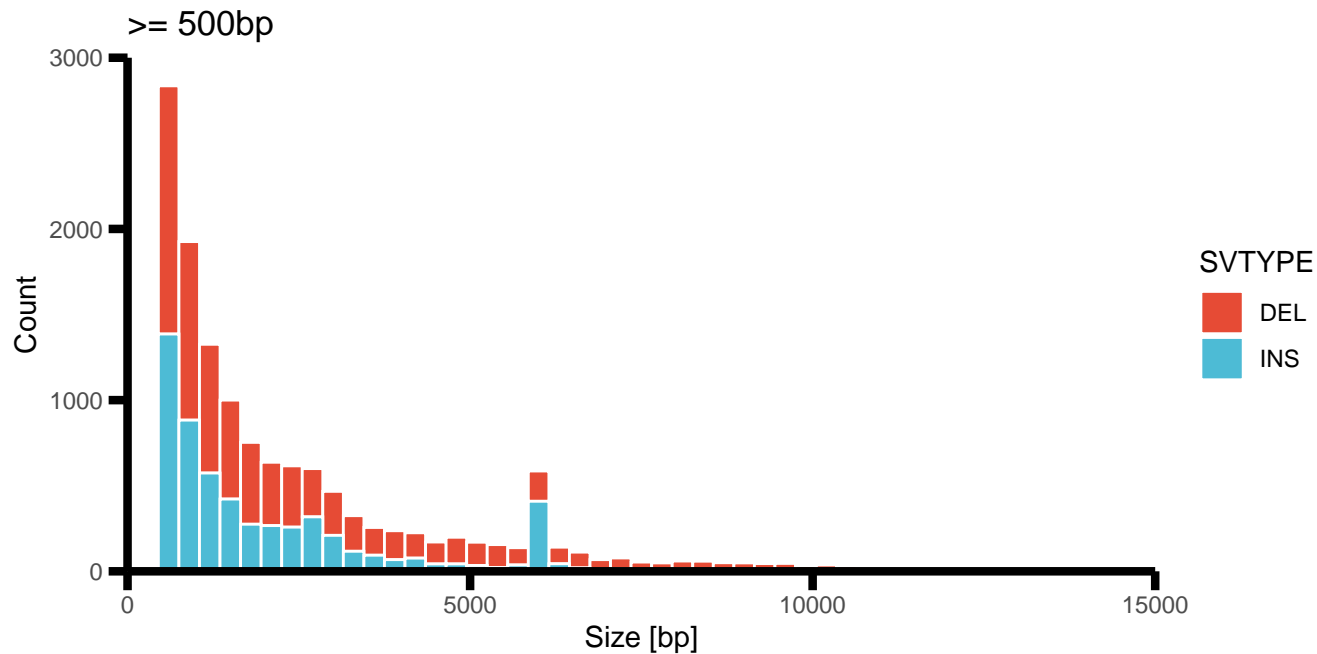

Supplement: Supplementary file 9 — Supplementary Data 3 [file 42003_2022_3953_MOESM9_ESM.zip › SuppData3/fig2-e.pdf]

type3   ● Intergenic   ● Intron   ● Exon   ● CDS

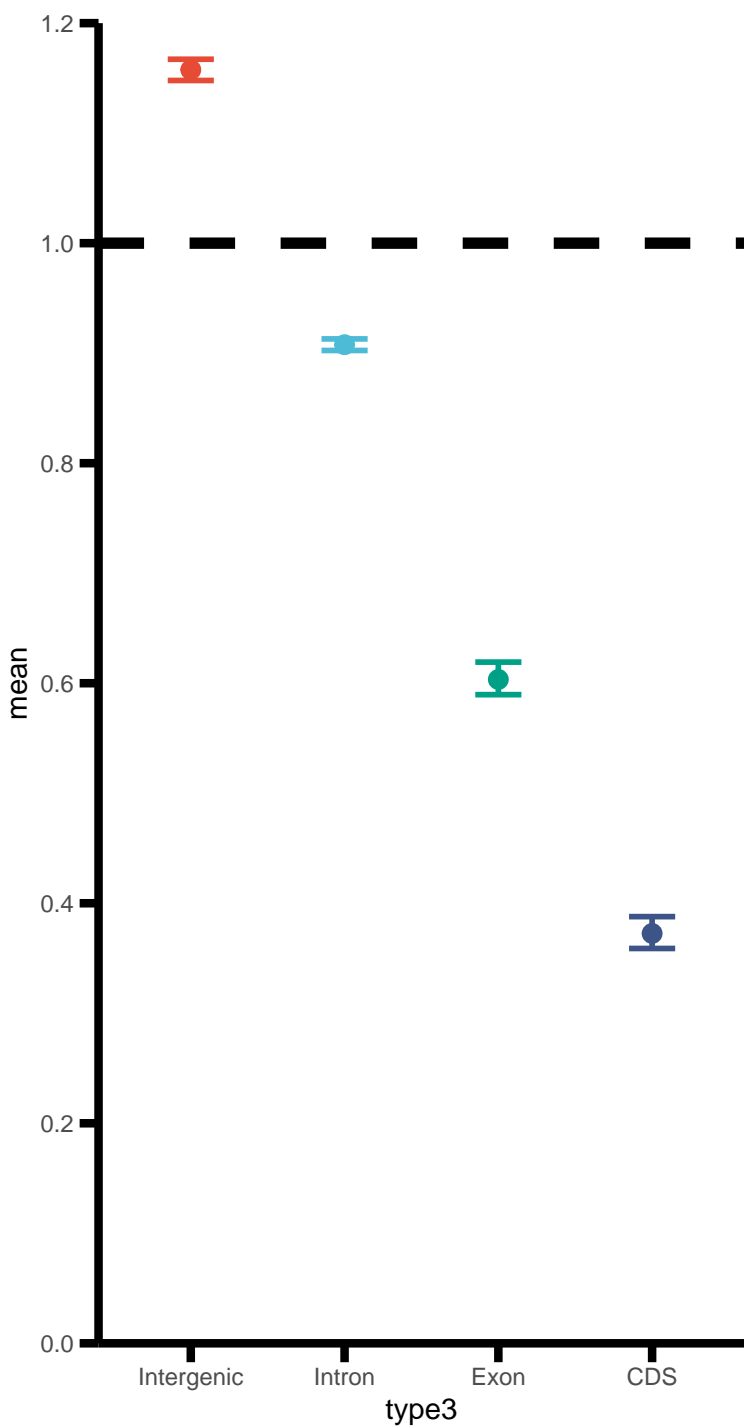

Supplement: Supplementary file 9 — Supplementary Data 3 [file 42003_2022_3953_MOESM9_ESM.zip › SuppData3/fig5-d.pdf]

N50 v.s. Insertion mean length (correlation: 0.801)

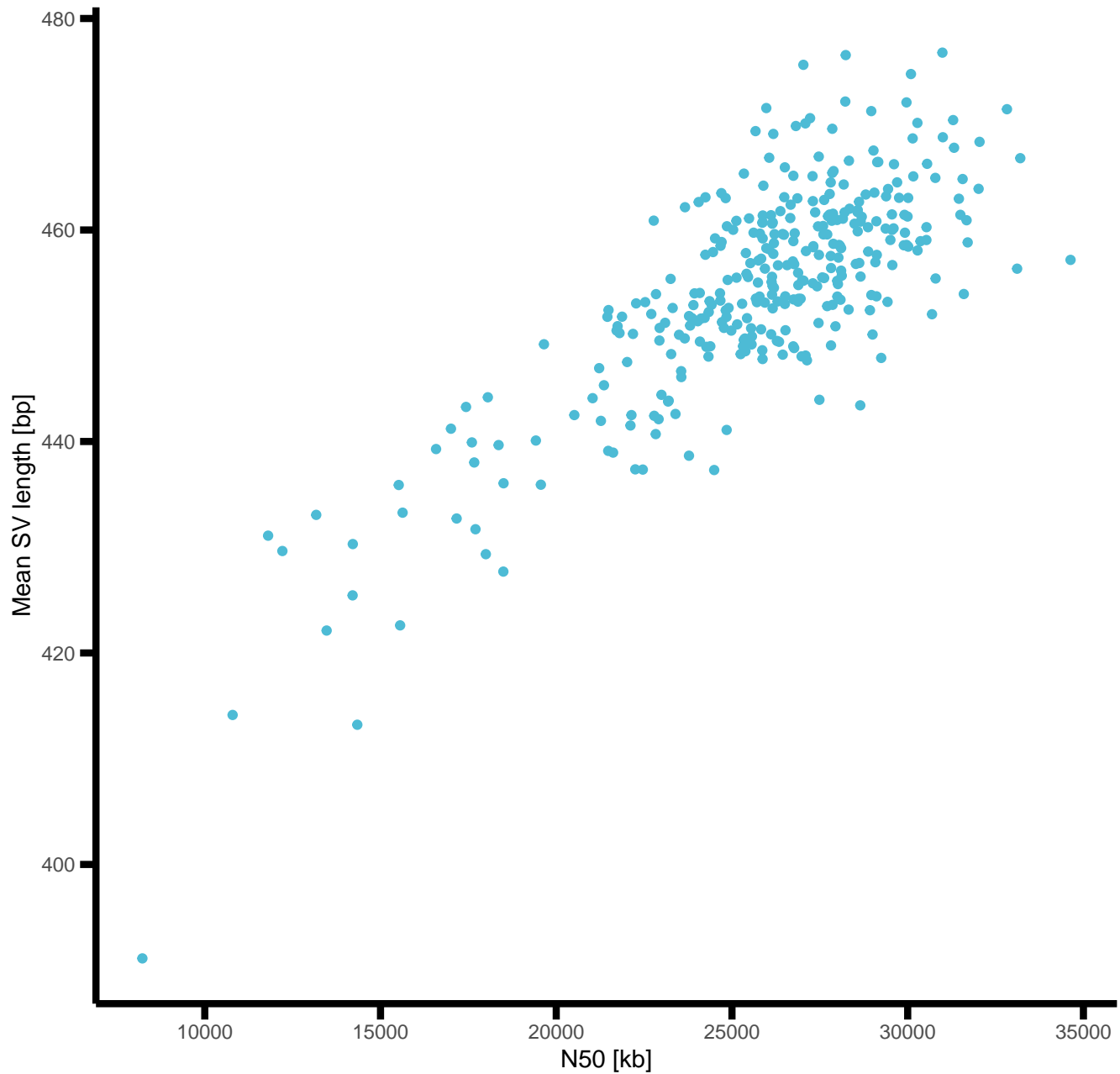

Supplement: Supplementary file 9 — Supplementary Data 3 [file 42003_2022_3953_MOESM9_ESM.zip › SuppData3/fig2-f-ins.pdf]

ToMMo\_MAF.CLASS.f

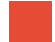

Singleton

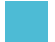

Rare

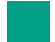

Low

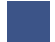

Common

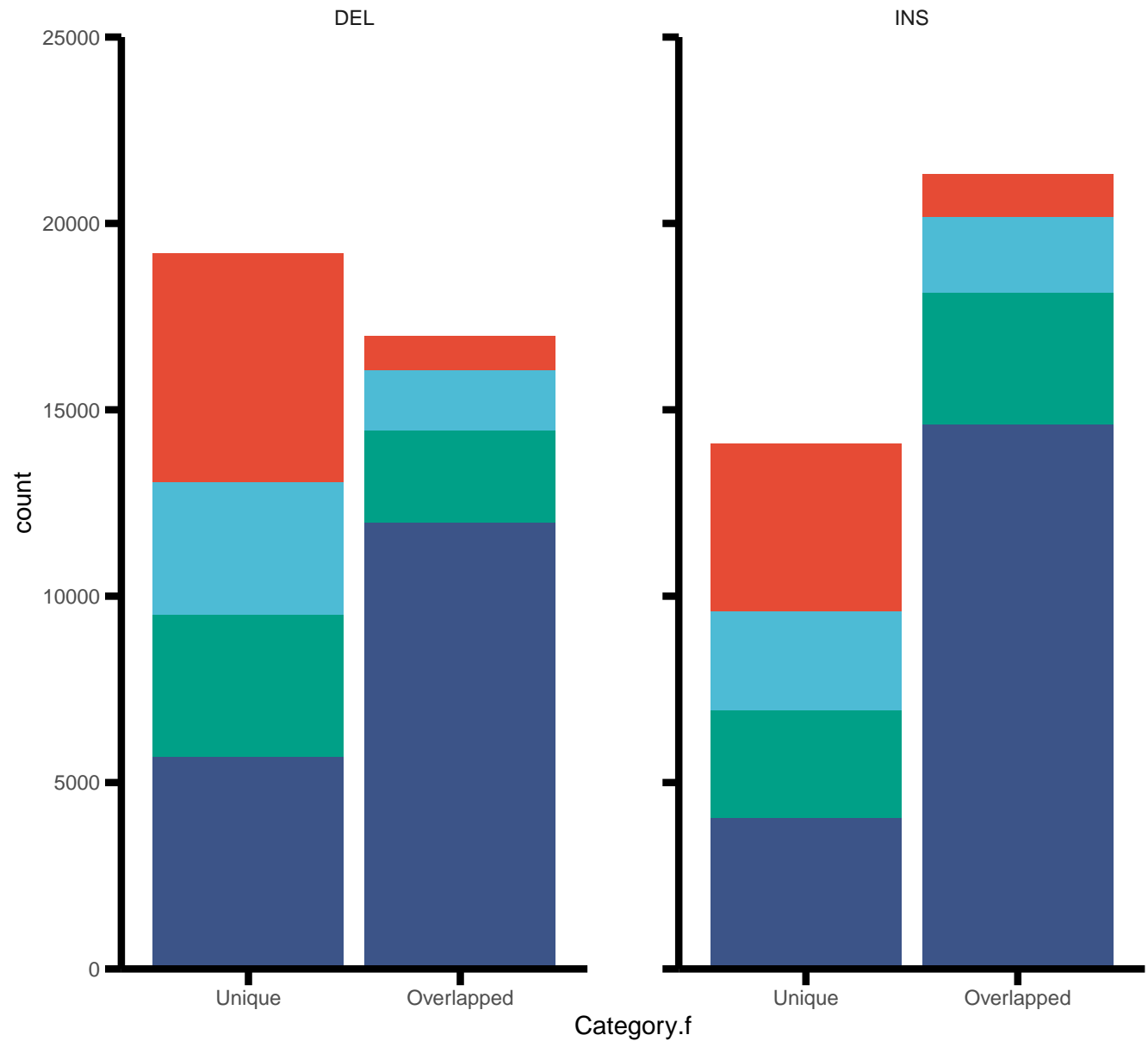

Supplement: Supplementary file 9 — Supplementary Data 3 [file 42003_2022_3953_MOESM9_ESM.zip › SuppData3/fig3-d.pdf]

INFO.SVTYPE DEL INS

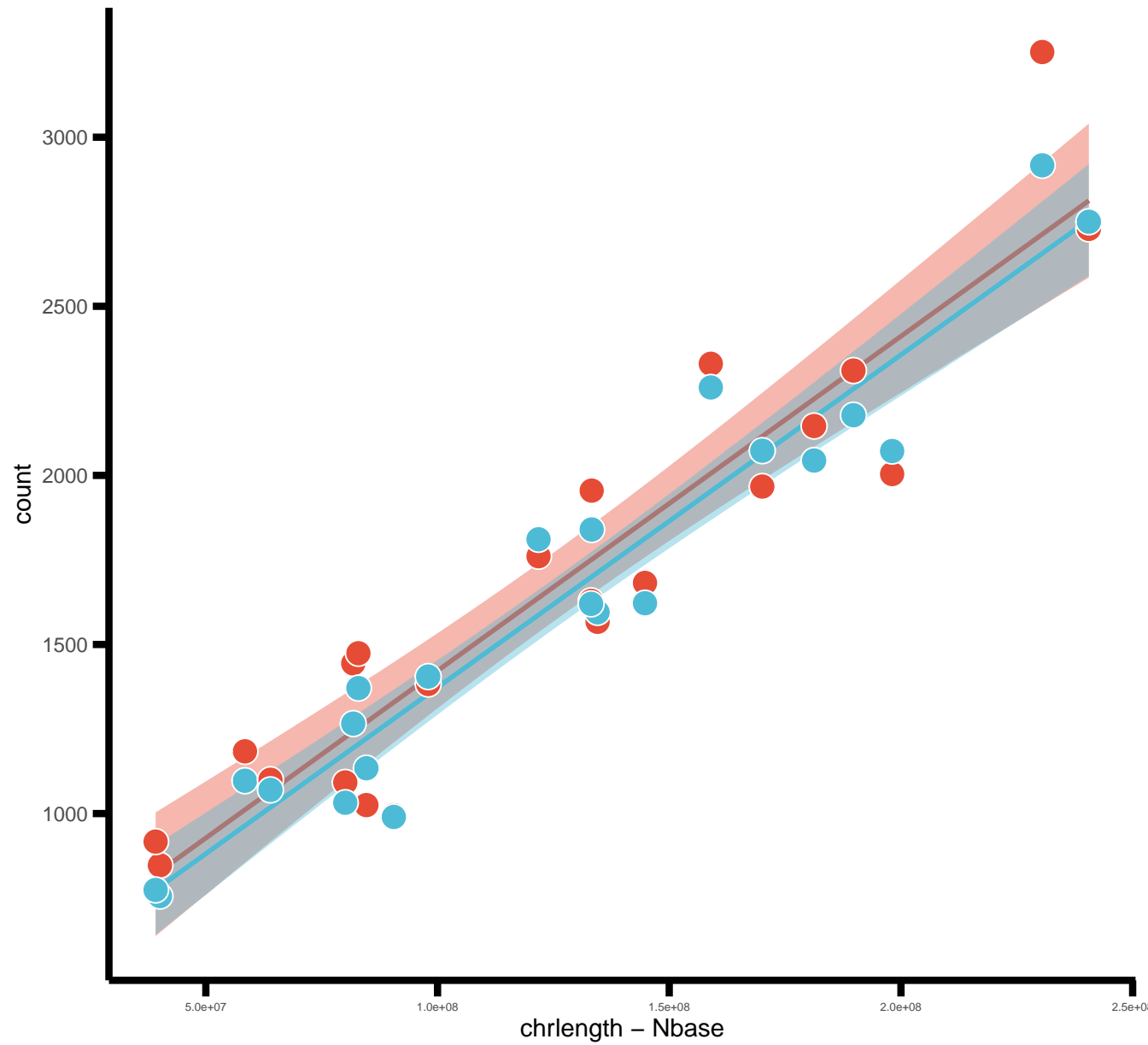

INFO.SVTYPE

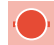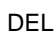

DEL

INS

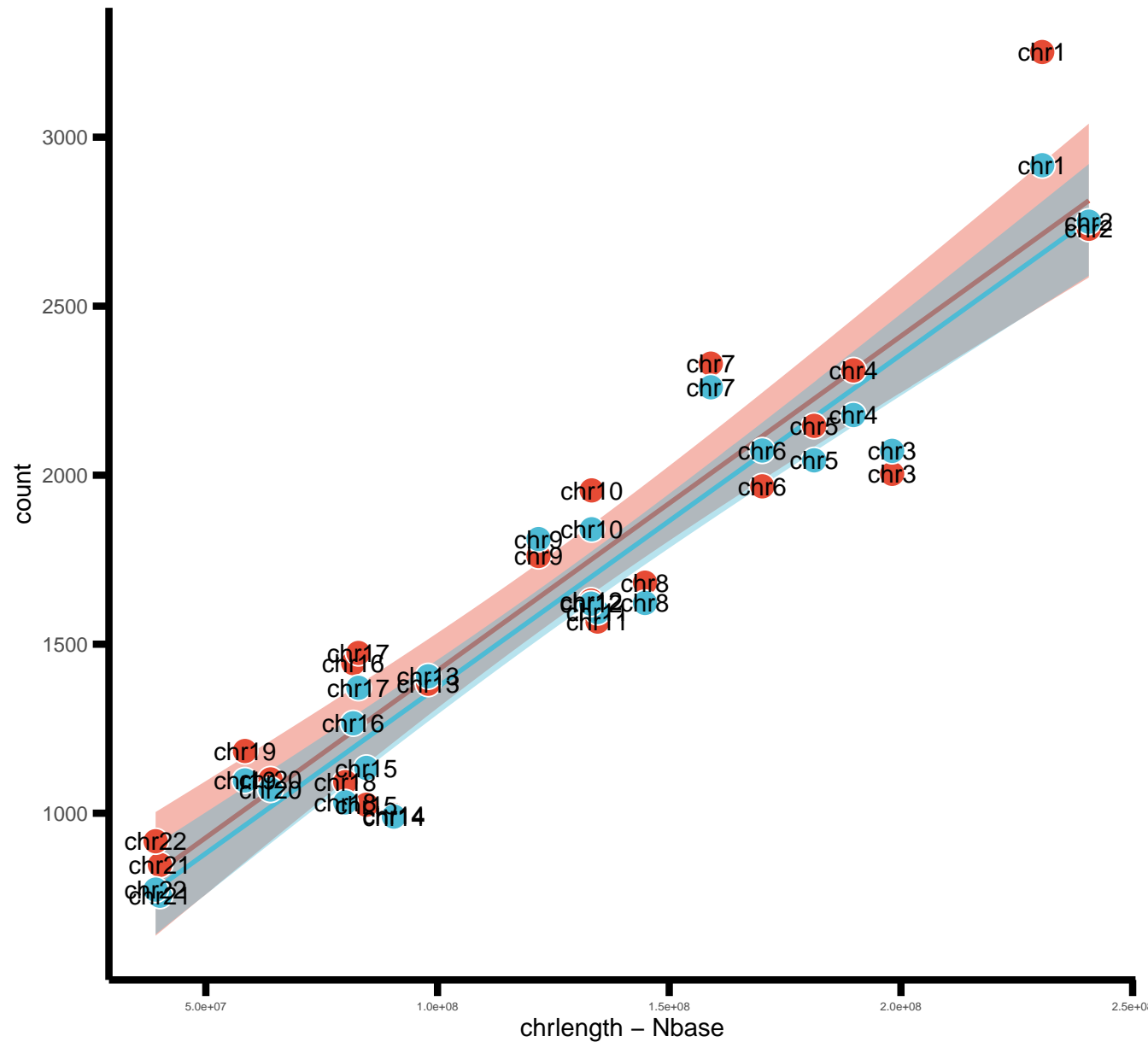

Supplement: Supplementary file 9 — Supplementary Data 3 [file 42003_2022_3953_MOESM9_ESM.zip › SuppData3/fig5-a.pdf]

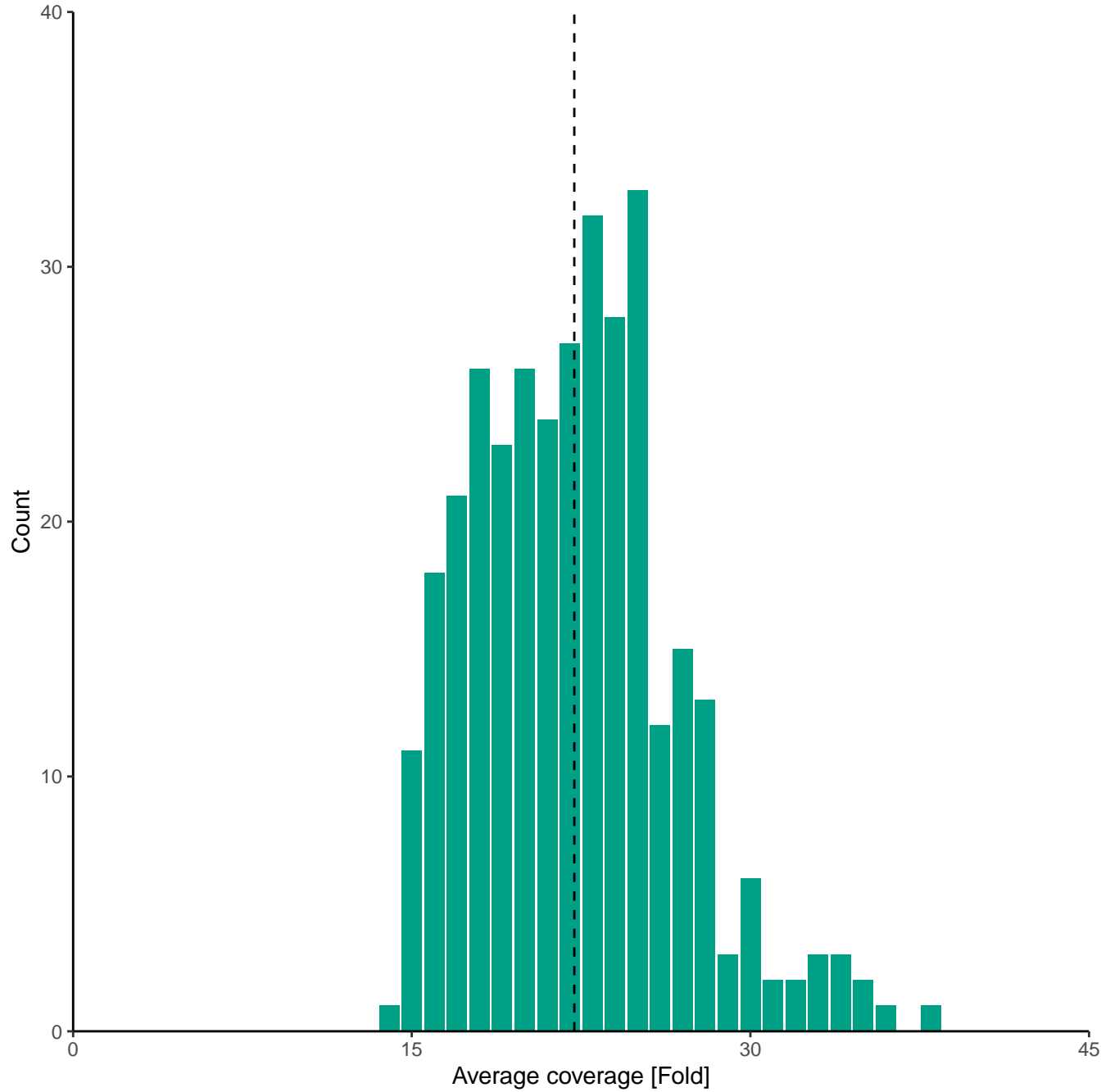

Supplement: Supplementary file 9 — Supplementary Data 3 [file 42003_2022_3953_MOESM9_ESM.zip › SuppData3/fig2-c.pdf]

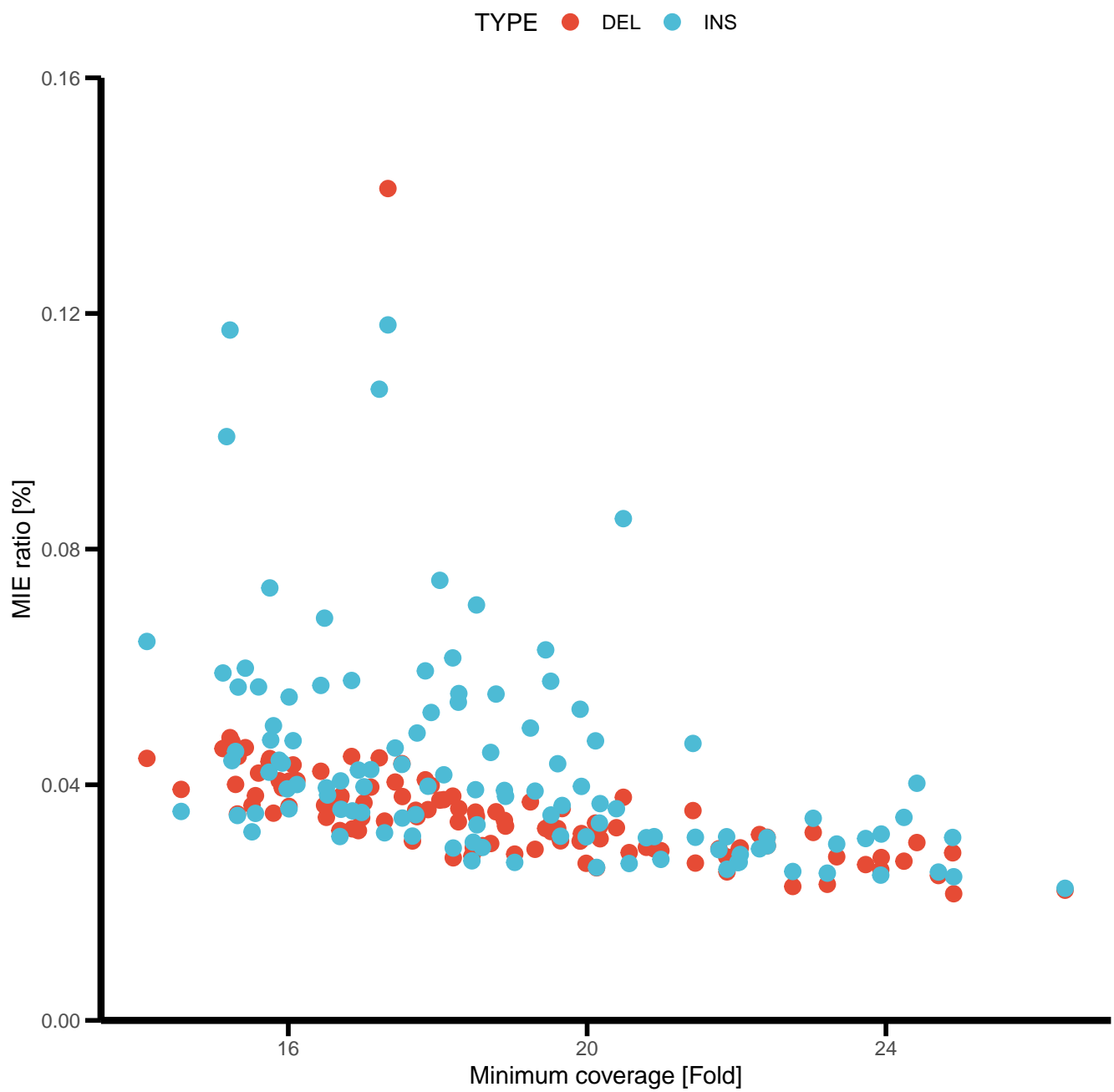

Supplement: Supplementary file 9 — Supplementary Data 3 [file 42003_2022_3953_MOESM9_ESM.zip › SuppData3/fig4-b.pdf]

Only overlapped SVs (INS)[correlation: 0.40]

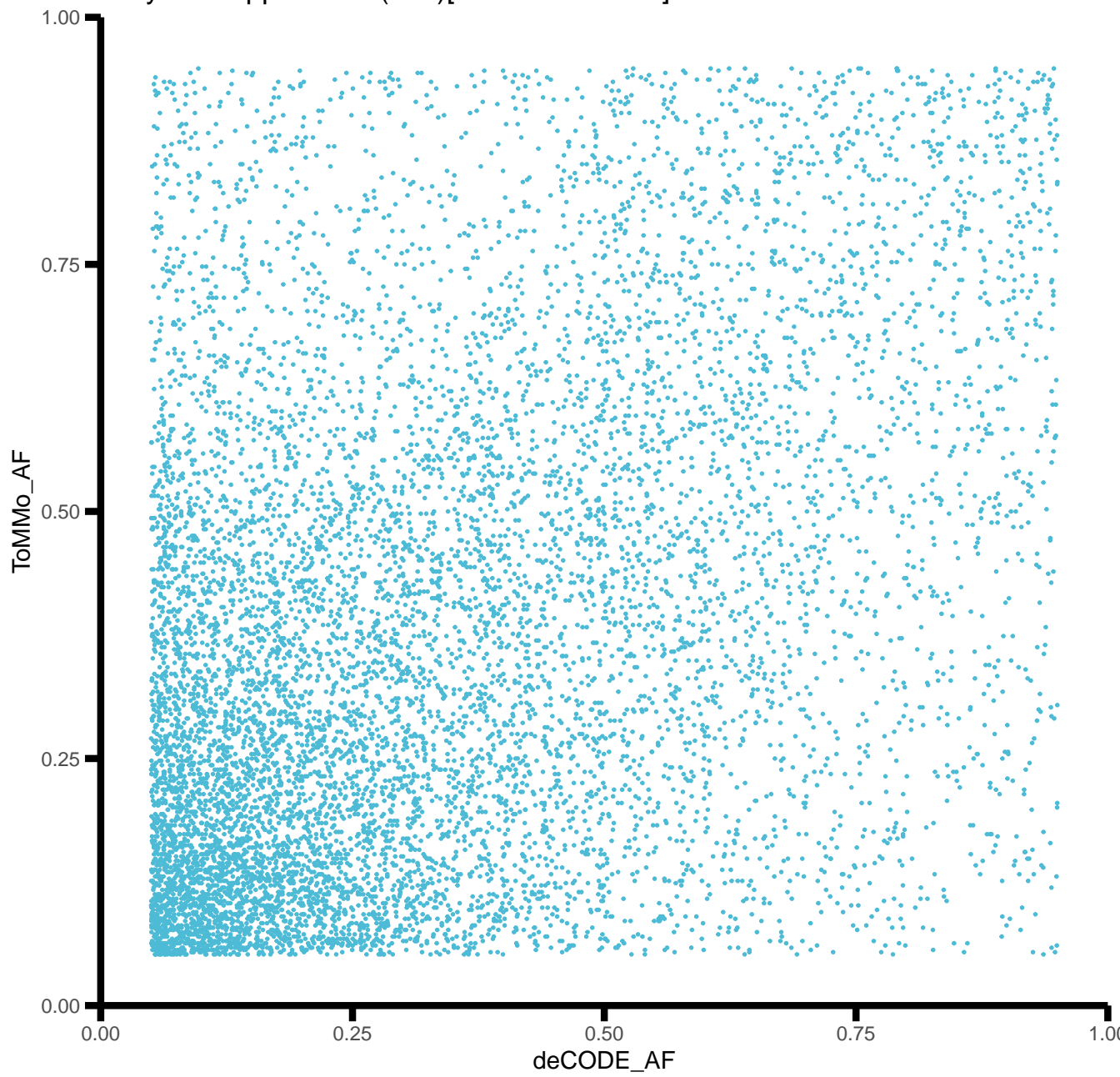

Only overlapped SVs (DEL)[correlation: 0.50]

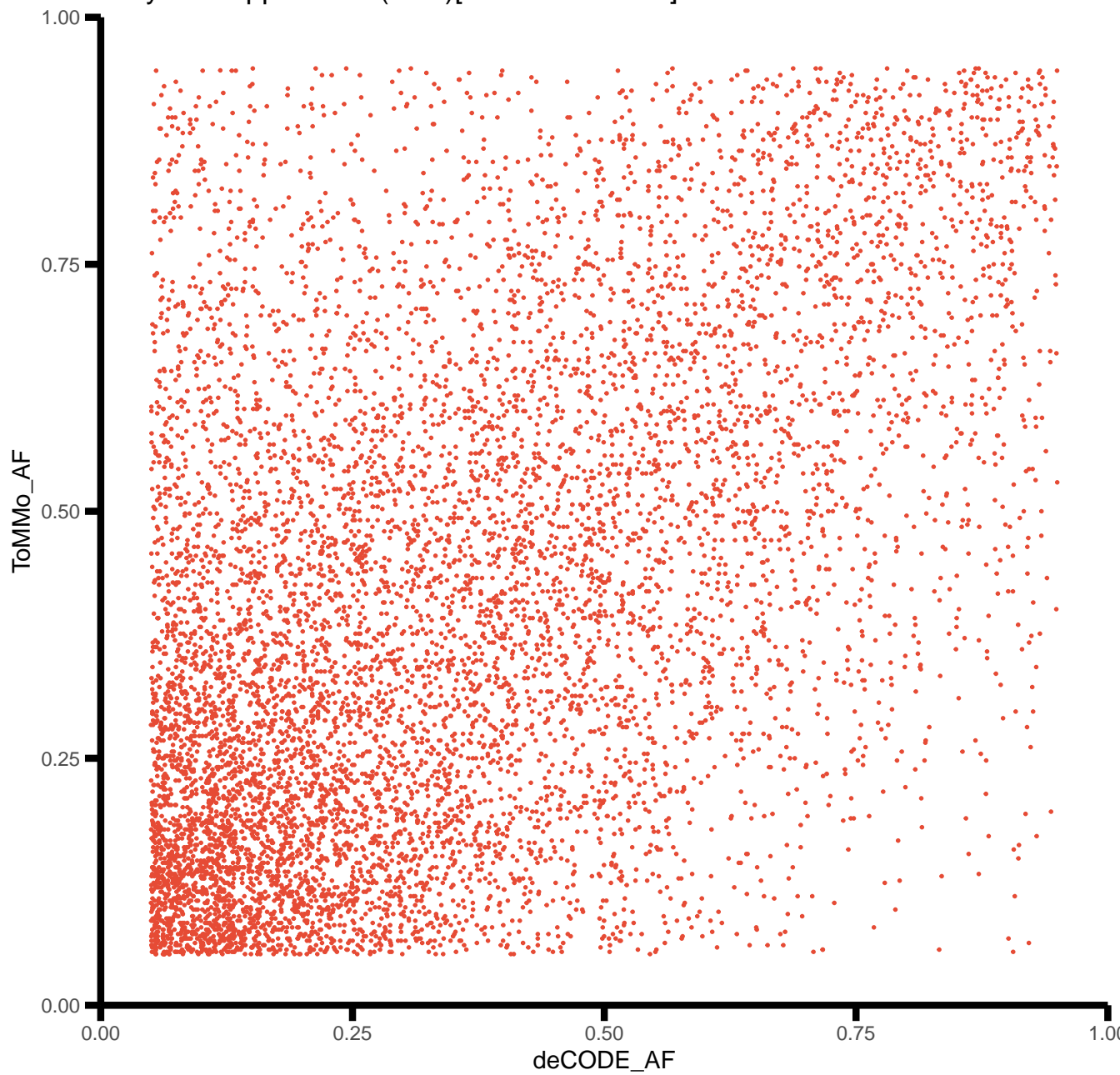

Supplement: Supplementary file 9 — Supplementary Data 3 [file 42003_2022_3953_MOESM9_ESM.zip › SuppData3/fig3-e.pdf]

N50 v.s. Deletion mean length (correlation: 0.537)

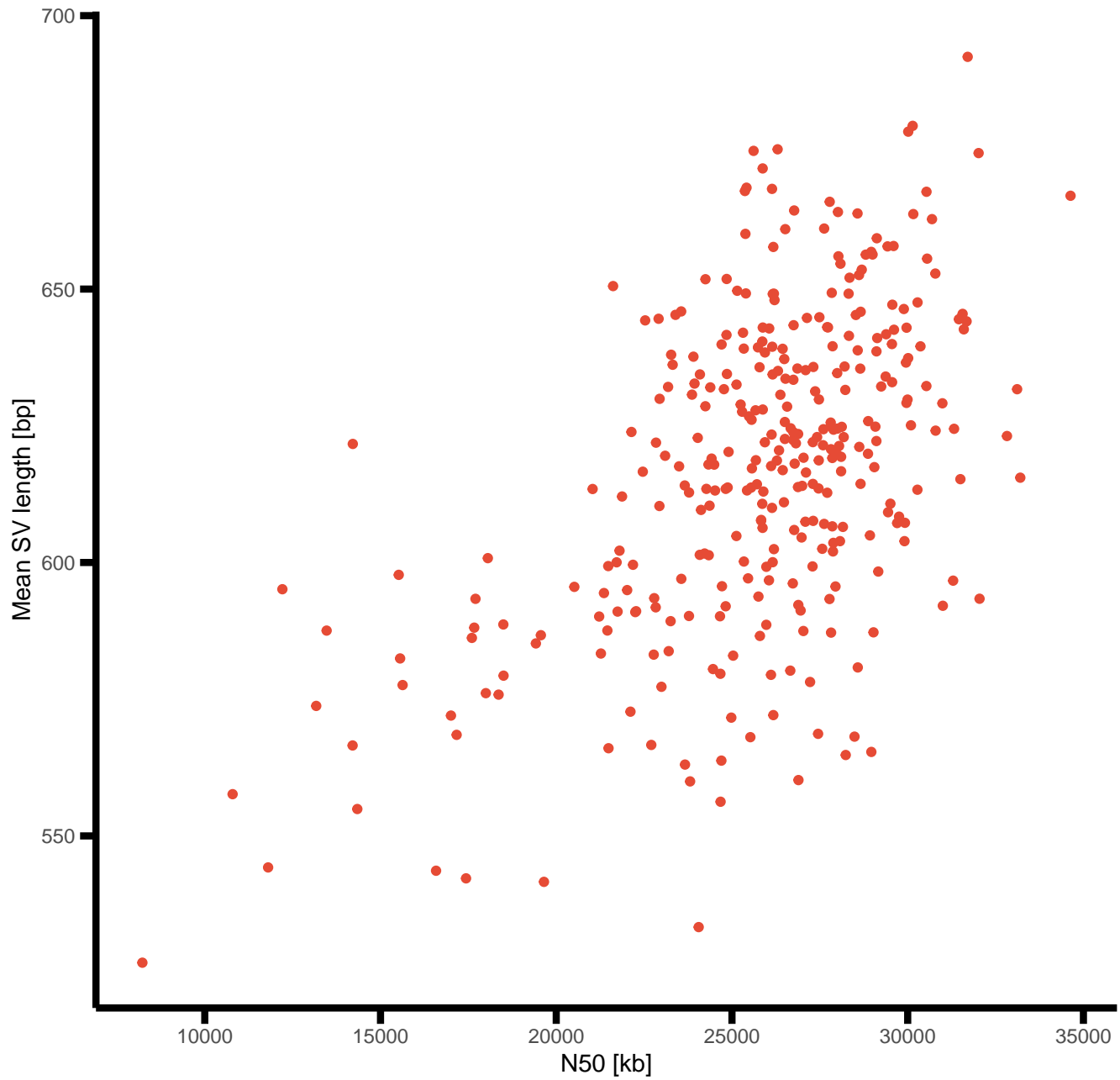

Supplement: Supplementary file 9 — Supplementary Data 3 [file 42003_2022_3953_MOESM9_ESM.zip › SuppData3/fig2-f-del.pdf]
